# How do patients with Parkinson’s disease and cerebellar ataxia read aloud? -Eye–voice coordination in text reading

**DOI:** 10.3389/fnins.2023.1202404

**Published:** 2023-08-11

**Authors:** Yasuo Terao, Shin-ichi Tokushige, Satomi Inomata-Terada, Tai Miyazaki, Naoki Kotsuki, Francesco Fisicaro, Yoshikazu Ugawa

**Affiliations:** ^1^Department of Neurology, Graduate School of Medicine, University of Tokyo, Tokyo, Japan; ^2^Department of Medical Physiology, Kyorin University, Mitaka, Japan; ^3^Department of Neurology, Kyorin University, Mitaka, Japan; ^4^Department of Human Neurophysiology, Fukushima Medical University, Fukushima, Japan

**Keywords:** eye-voice coordination, eye tracking, Parkinson’s disease, spinocerebellar degeneration, reading

## Abstract

**Background:**

The coordination between gaze and voice is closely linked when reading text aloud, with the gaze leading the reading position by a certain eye–voice lead (EVL). How this coordination is affected is unknown in patients with cerebellar ataxia and parkinsonism, who show oculomotor deficits possibly impacting coordination between different effectors.

**Objective:**

To elucidate the role of the cerebellum and basal ganglia in eye–voice coordination during reading aloud, by studying patients with Parkinson’s disease (PD) and spinocerebellar degeneration (SCD).

**Methods:**

Participants were sixteen SCD patients, 18 PD patients, and 30 age-matched normal subjects, all native Japanese speakers without cognitive impairment. Subjects read aloud Japanese texts of varying readability displayed on a monitor in front of their eyes, consisting of Chinese characters and hiragana (Japanese phonograms). The gaze and voice reading the text was simultaneously recorded by video-oculography and a microphone. A custom program synchronized and aligned the gaze and audio data in time.

**Results:**

Reading speed was significantly reduced in SCD patients (3.53 ± 1.81 letters/s), requiring frequent regressions to compensate for the slow reading speed. In contrast, PD patients read at a comparable speed to normal subjects (4.79 ± 3.13 letters/s vs. 4.71 ± 2.38 letters/s). The gaze scanning speed, excluding regressive saccades, was slower in PD patients (9.64 ± 4.26 letters/s) compared to both normal subjects (12.55 ± 5.42 letters/s) and SCD patients (10.81 ± 4.52 letters/s). PD patients’ gaze could not far exceed that of the reading speed, with smaller allowance for the gaze to proceed ahead of the reading position. Spatial EVL was similar across the three groups for all texts (normal: 2.95 ± 1.17 letters/s, PD: 2.95 ± 1.51 letters/s, SCD: 3.21 ± 1.35 letters/s). The ratio of gaze duration to temporal EVL was lowest for SCD patients (normal: 0.73 ± 0.50, PD: 0.70 ± 0.37, SCD: 0.40 ± 0.15).

**Conclusion:**

Although coordination between voice and eye movements and normal eye-voice span was observed in both PD and SCD, SCD patients made frequent regressions to manage the slowed vocal output, restricting the ability for advance processing of text ahead of the gaze. In contrast, PD patients experience restricted reading speed primarily due to slowed scanning, limiting their maximum reading speed but effectively utilizing advance processing of upcoming text.

## Introduction

Speech production involves the coordination of various motor activities, such as respiration, phonation, articulation, resonance, and prosody (Fabbri et al., 2017; Dashtipour et al., 2018). Voice disorders refer to conditions that affect the production or quality of the voice, impacting pitch, loudness, resonance, and overall voice quality. Speech disorders, on the other hand, involve difficulties in producing speech sounds or using language effectively, affecting articulation, fluency, or voice quality during speaking. Neurological disorders like cerebellar ataxia and parkinsonism can involve both aspects of vocal output disorder (Suppa et al., 2015, 2020).

When reading aloud, eye movements synchronize with the actions of the vocal organs to gather visual information from the text. This information undergoes lexical and phonological processing and is then converted into spoken words through articulation and vocal output production. The coordination between eye movements and vocal output during oral reading is known as “eye-voice coordination.” Studying gaze movement during oral reading helps us understand the process of converting written words into spoken language.

Typically, when reading aloud, individuals focus their gaze slightly ahead of the current word being spoken (referred to as eye-voice lead [EVL] or eye–voice span [EVS]). This means there are differences between the words they fixate on and the words they pronounce. The gaze input is used to visually process upcoming letters in the text, converting them into lexical and phonological information. This information is temporarily stored in memory or the “verbal sketchpad” (De Luca et al., 1999, 2002, 2013; Rayner, 2009; Baddeley and Hitch, 2019), while the acquired information is simultaneously processed and transformed into speech output for vocal output/verbal expression.

Early studies by Buswell (1921) and Fairbanks (1937) showed that the gaze position over the text precedes the position read by the voice by approximately 10–15 letters in space (spatial EVL) or 0.5–1 s in time (temporal EVL). These studies relied on the “light-off” method, which estimated the preceding gaze based on the number of words that could be articulated after the room or monitor light was turned off. However, they did not directly record eye movements and instead made speculations as to the amount of letters processed by preceding gaze.

Recent advancements in eye-tracking technology have allowed us to gain a better understanding of the precise coordination between eye movements and vocalization in normal individuals. By recording both voice and eye movements simultaneously, researchers have been able to examine eye-voice coordination in normal subjects and shed light on the underlying pathophysiological processes in reading disabilities like dyslexia.

De Luca et al. (2013) conducted a study in which they had native Italian speakers with dyslexia read Italian texts aloud and compared their performance to that of normal subjects. They found that dyslexic readers exhibited slower reading compared to control peers. Dyslexic readers showed more silent pauses, sounded-out behaviors, and slightly longer word articulation times. Additionally, they showed reduced EVL compared to normal subjects. Similarly, individuals with autistic spectrum disorder (ASD) also displayed reduced EVL when asked to quickly name numbers arranged in rows (rapid automatic naming tasks, RAN). This reduction in EVL was associated with slower naming and indicated a reduced capacity of the “verbal sketchpad” in these individuals (Zoccolotti et al., 2013; Hogan-Brown et al., 2014). These findings suggest that reduced EVL reflects a diminished capacity of the “verbal sketchpad” in these patients, leading to less efficient reading.

In individuals without reading difficulties, enhanced eye-voice span (EVL) has been linked to increased automation in reading skills, such as proficient reading and faster reading speed or word naming. How does the length of EVL contribute to faster or more automatic reading? The eyes tend to move ahead of the articulatory system because visual processing is quicker than processing the articulation of the perceived word (Laubrock and Kliegl, 2015). One advantage of the eyes leading the voice is that the longer interval between the eyes and the voice aids in reading faster or more automatically. In typically developing individuals, extensive practice establishes automaticity in reading-related skills, such as in RAN, by reducing the need for attentional control and freeing up various attentional processes, such as working memory. However, Inhoff argues that the eyes do not necessarily have to move ahead of the voice but can instead wait until the voice catches up, especially when reading at a very slow pace (Inhoff et al., 2011; Laubrock and Kliegl, 2015; Silva et al., 2016).

If the reader uses the initial gaze time on the text ahead of the currently uttered word or syllable, the time can be used to finish the processing of the currently gazed part of the text as well, that is, parallel processing of the two components (or words) in the text can take place at the same time (Jones et al., 2008, 2016; Protopapas et al., 2013; Laubrock and Kliegl, 2015). Additionally, buffering of the material that can be rapidly decoded and translated from graphemic input into a phonological code also allows faster reading through processing of word articulation into chunks. Thus, longer EVL helps subjects read automatically or faster, whereas subjects can read faster by “stretching” the EVL while reading. Shorter EVL in individuals with dyslexia or ASD can disrupt automaticity in language-related skills as the diminished EVL hampers parallel processing (Silva et al., 2016).

Few studies to date have examined the impact of impaired oculomotor control on reading aloud (Schattka et al., 2010; Stock et al., 2020). Reading difficulties during oral reading are often observed in individuals with neurological disorders like Parkinsonism and cerebellar ataxia. The coordination between eye movements and voice involves multiple brain regions, particularly the cerebellum, which controls the movements of these two components (Miall and Jenkinson, 2005; Nitschke et al., 2005; Stoodley and Stein, 2013; Modroño et al., 2020; Rizzo et al., 2020). Therefore, cerebellar pathology can impact this coordination associated with deficits in oculomotor control.

Basal ganglia disorders can impact gaze movement during reading in a distinct manner. Individuals with Parkinson’s disease (PD) often experience various abnormalities in their eye movements while reading. These include an increased number of both forward and backward eye movements (saccades) and longer periods of fixation, leading to a slower scanning of the text (Stock et al., 2020).

In PD, the basal ganglia excessively inhibit the oculomotor system, causing the amplitude of eye movements while scanning the text to be smaller (hypometric) compared to individuals without the disorder. Additionally, the frequency of these eye movements per unit of time also decreases (Terao et al., 2011; 2013; Matsumoto et al., 2011a,b; Tokushige et al., 2018). As a result, the speed at which the gaze scans the text is slower, leading to a decrease in reading speed (Waldthaler et al., 2018).

No spaces are usually inserted between words in Japanese, this may make Japanese reading an exceptional case as opposed to Western and other languages. However, reading in the RAN context (with space intervals) and those reading a normal text represents a different context in terms of reading, with the latter more emphasis for eye movements (eye jumps between words). Here we focused on the overall time course of natural, continuous reading of Japanese instead of discrete fixations on each word.

This study aimed to explore the impact of gaze on natural and continuous oral reading (reading aloud) in individuals with Parkinsonism and cerebellar ataxia. By investigating the role of gaze in this specific reading behavior, we aimed to shed light on the underlying pathophysiology. Although silent reading has received more attention in research, this coordination is crucial even in silent reading, as it involves subvocalization as a latent output. Here, we conducted a study to examine the coordination between eye movements and voice in native Japanese patients with Parkinson’s disease (PD) as they read Japanese text of varying readability. We compared their performance with that of age-matched healthy individuals and patients with spinocerebellar degeneration (SCD). The participants read Japanese texts displayed on a monitor screen, and we analyzed the coordination between their gaze and voice while reading. We simultaneously recorded and tracked their eye movements and voice utterances to assess the fundamental relationship between eye and voice during reading. Additionally, we investigated how the subjects adjusted their eye movements, voice latency, and reading speed in response to changes in the text’s readability according to the demands of the text.

## Methods

### Subjects

Subjects were native Japanese speakers. Study participants were 16 SCD patients (11 males, 5 females; age 62.2 ± 9.5 years); 9 multiple system atrophy cerebellar-type (MSAC), 5 spinocerebellar ataxia (SCA6 4, SCA31, unknown 1) and 18 PD patients (11 males, 8 females, age 70.1 ± 4.4 years). The patients were recruited at the outpatient and inpatient sections of the University of Tokyo Hospital. Initially, 18 SCD patients were recruited, but two subjects were excluded because of poor recording. Thirty age-matched normal subjects (11 males, 17 females, age 70.8 ± 3.7 years) were also recruited to obtain age-matched control data. The following experiments were conducted according to the declaration of Helsinki, after obtaining written informed consent from the subjects. The procedures of the experiment were approved by the local ethical committee [Reference number: 2411-(10)]. None of the subjects had mental or cognitive problems, and all had a Mini-Mental State Examination score above 25. There were no dysmorphisms or paramorphisms in the subjects.

The inclusion criteria of SCD patients were adult patients with MSAC and SCD patients with predominant cerebellar manifestation, and the exclusion criteria were those with hearing loss that interfered with verbal communication, severe dysarthria that prevented the subjects from performing the reading task, or severe cognitive impairment that they prevented them from understanding or performing the task procedure, or severe orthostatic hypotension that they cannot keep seated for 1 h.

All SCD patients presented predominantly with cerebellar ataxia with minimal parkinsonism. Among them, the diagnosis of MSA was based on Gilman’s criteria (Gilman et al., 1999, 2008). Selection of other SCD patients in this study was based on pure progressive cerebellar symptoms throughout the follow-up period but no brainstem involvement, as also demonstrated by cerebellar atrophy and preservation of brainstem in neuroimaging. The disease stage of SCD patients was assessed according to the ataxia scale of Schmitz-Hübsch et al. (2006), as in our previous studies (Terao et al., 2016, 2017). Stage 0 represents no gait difficulty, whereas stage 1 represents patients at disease onset, as defined by onset of gait difficulties. At stage 2, patients lose their independent gait, necessitating the use of a walking aid or a supporting arm to walk. At stage 3, patients are permanently confined to a wheelchair. Stages in between were given intermediate scores (such as 2.5).

PD patients were diagnosed according to the British Parkinson’s Disease Society Brain Bank Criteria. H-Y stage of the patients was 1.6 ± 0.9 on average. The disease stage of motor symptoms was 14.9 ± 1.9 on average as assessed by means of the Unified Parkinson’s Disease Rating Scale (UPDRS) Scale motor score (UPDRS-III). Subject information is summarized in Table 1. None of the patients received DBS surgery. PD patients continued to take their medication as usual, and were examined approximately 3–4 h after drug intake in the morning, which would minimize the effects on eye movements according to our previous study (Yugeta et al., 2008). Levodopa equivalent dose was 321.3 ± 131.9 mg on average. For SCD patients, the drug patients took was taltirelin hydrate in most cases, while some patients were taking clonazepam, L-threo-DOPS, midodrine hydrochloride, diphenidol hydrochloride as drugs potentially acting on the central nervous system. L-dopa or other dopaminergic drugs were not taken by any of the SCD patients.

**Table 1:** Details of included subjects.

Diagnosis	No. of cases	Male	Female	Age (yrs)	Duration (yrs)	Disease stage*	H-Y stage	UPDRS motor score	LEDD (mg)
Normal	30	13	17	70.8 ± 3.7	–	–		–	
SCD	16	11	5	62.1 ± 9.5	7.3 ± 5.6	1.7 ± 0.7			
PD	18	11	7	70.1 ± 4.3	3.8 ± 0.8	–	1.6 ± 0.9	14.9 ± 1.9	321.3 ± 131.9

*Stage 0 represents no gait difficulty, whereas Stages 1 represent patients at disease onset, as defined by onset of gait difficulties. At Stage 2, patients lose independent gait, and needs a walking aid or a supporting arm to walk with. At Stage 3, patients come to be permanently confined to a wheel chair. Stages in between were scored intermediate (such as 2.5).

### Task procedure

**Figure 1:**
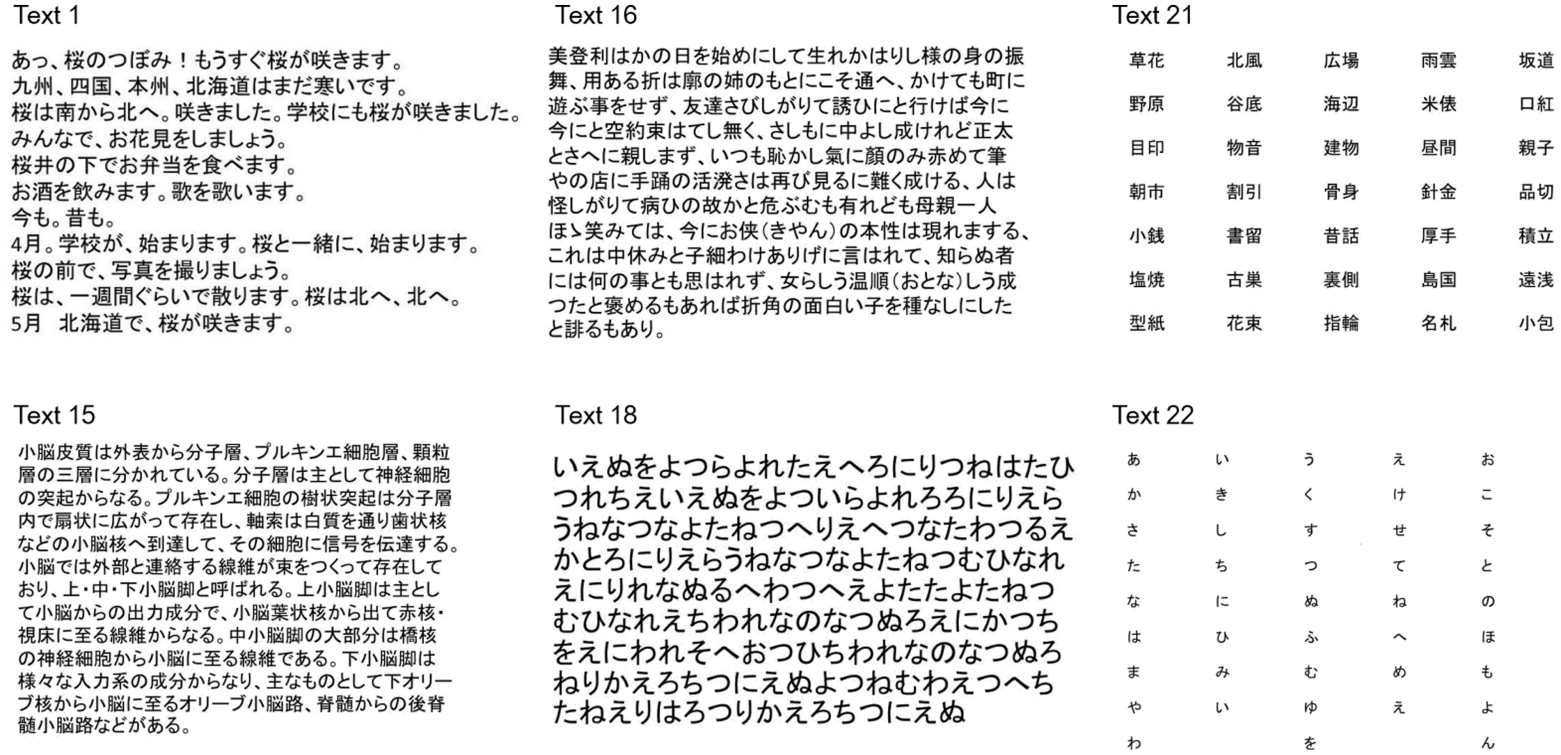
Examples of texts presented in the study. **Text 1**: Transcript of Japanese conversation, entry level (entry level, word level 350), **Text 15**: Japanese medical textbook (word level 2000), **Text 16**: Japanese pre-modern literature (word level 2000), **Text 18**: random sequence of Japanese phonograms (hiragana), **Text 21**: arranged list of words (Kanji compounds), **Text 22**: Japanese syllabary arranged from top to bottom in each column and from left to right columns.

Subjects read aloud Japanese texts (Table 2 and Figure 1) presented on a 17-inch monitor screen with a refresh rate of 60 Hz (Dell E173FPb, Dell, Kawasaki, Japan, screen resolution 1024 × 768). Head movement was restricted by the chin and forehead rests of the eye tracker, although some slight movements were noted when the subjects read the text aloud. The monitor was positioned vertically at a viewing distance of 50 cm in front of their eyes.

**Table 2:** Text used in the study.

Text number	Vocabulary level in Japanese	Readability level	Font size
Text1	350	Entry level	24
Text2		Elementary level (low grades)	24
Text3	500	Elementary level (high grades)	24
Text4	800	Pre-intermediate	24
Text5		Pre-intermediate	32
Text6		Pre-intermediate	32
Text7	1000	Intermediate level	24
Text8		Intermediate level	32
Text9		Intermediate level	32
Text10		Intermediate level	32
Text11		Intermediate level	16
Text12		Intermediate level	16
Text13		Intermediate level	16
Text14	2000	Japanese modern literature	16
Text15	Above 2000	Extracts from medical textbooks	24
Text16		Japanese premodern writings	32
Text17	–	Random sequence of Japanese ancient writings	30
Text18	–	Random sequence of Japanese phonograms	24
Text19	–	Random sequence of Japanese phonograms	16
Text20	–	Arranged list of words (hiragana words)	24
Text21	–	Arranged list of words (kanji compounds)	24
Text22	–	Japanese syllabary (list of hiragana phonograms)	24

Vocabulary level in Japanese, text readability and font sizes of each text used in the present study. Texts 1–19 are typical Japanese texts, without spaces between words, and are arranged in order of decreasing readability (from easier to more difficult to read) in this table. Texts 20–22 are arranged word lists (texts 20, 21): arranged list of Kanji words (Kanji compounds), with spaces in between, or Japanese syllabary (text 22).

The subjects had to read the text clearly at their natural and comfortable speed and usual prosody. The eye movement data was recorded from the dominant eye, although when recording from the dominant eye was not stable, the non-dominant eye was recorded instead. The gaze location on the screen was measured simultaneously by a video-based eye tracking system (EyeLink 1000, SR Research, Mississauga, Ontario, Canada) at a sampling rate of 1000 Hz, and spatial resolution of less than 0.04°. The output from the microphone was fed to a PC a short-latency ASIO driver that connected the microphone input directly to a USB audio interface (M-track M-audio, M-audio Japan, Tokyo, Japan) with a recording latency of 2–6 ms, and also interfaced to the eye tracker by the EyeLink Experiment Builder software. Eye movement data was fed to the EyeLink Host computer via an analog board, and was then fed to the Experiment builder computer through an ethernet cable.

Tasks were created using the SR Research Experiment Builder software, version 1.5.58. The gaze position on the monitor screen was recorded using a video-based eye tracking system (EyeLink 1000, SR Research, Mississauga, Ontario, Canada), while the voice simultaneously digitally recorded by a headset with a microphone. Prior to the experiments, the subjects performed a nine-point gaze calibration procedure to map the ocular fixation position onto screen coordinates. The calibration was considered to be valid if the maximum spatial error was less than 1° and the average error was less than 0.5°.

### Stimulus

Japanese texts of 5–14 lines in length, written in the horizontal direction from left to right with Chinese characters and Japanese phonetic lettering intermingled, were presented for 45 s each (Table 2 and Figure 1). Each letter subtended a visual angle of 0.6–1.3 degrees (0.85 cm on the screen, corresponding to approximately 1° of visual angle). Texts of various reading difficulty levels and letter sizes were used. Most of the texts presented were taken from those frequently adopted in Japanese textbooks. These texts were selected since they use standard Japanese text styles and are actually used for teaching Japanese to foreigners living in Japan (Hayashi and NPO Tadoku Supporters, 2012; Kio and NPO Tadoku Supporters, 2012; Koizumi and NPO Tadoku Supporters, 2012; Sakai and NPO Tadoku Supporters, 2014). The text levels reflecting the overall reading difficulty was defined according to the frequency of words and grammar structures included in the text. Correspondence to grades of reading levels in ordinary Japanese schools were made based on this information; the texts ranged in difficulty from the first grade of elementary school to the university/academic level. The two most difficult texts were taken from a Japanese medical textbook, in which the included medical terminology was unfamiliar to the subject, and from pre-modern Japanese writing, which was written in the style of the Edo-Meiji era (1700–1800), understandable to many contemporary Japanese people but using words and grammar of a pre-modern style, thus increasing the reading difficulty.

To account for the spaces between words that are present in Western languages, we also presented word lists in both hiragana phonograms and kanji graphemes, separated by spaces [text 20: arranged list of hiragana words, text 21: arranged list of kanji words (kanji compounds)]. Also, we presented the Japanese syllabary, separated by spaces and consisting of 50 different hiragana graphemes ordered in a sequence. This Japanese is learned at the beginning of school (Text 22), just as the alphabet is learned for Western languages. When reading this text, the subjects did not actually “read” it since they are expected to access it from memory.

### Data processing

**Figure 2:**
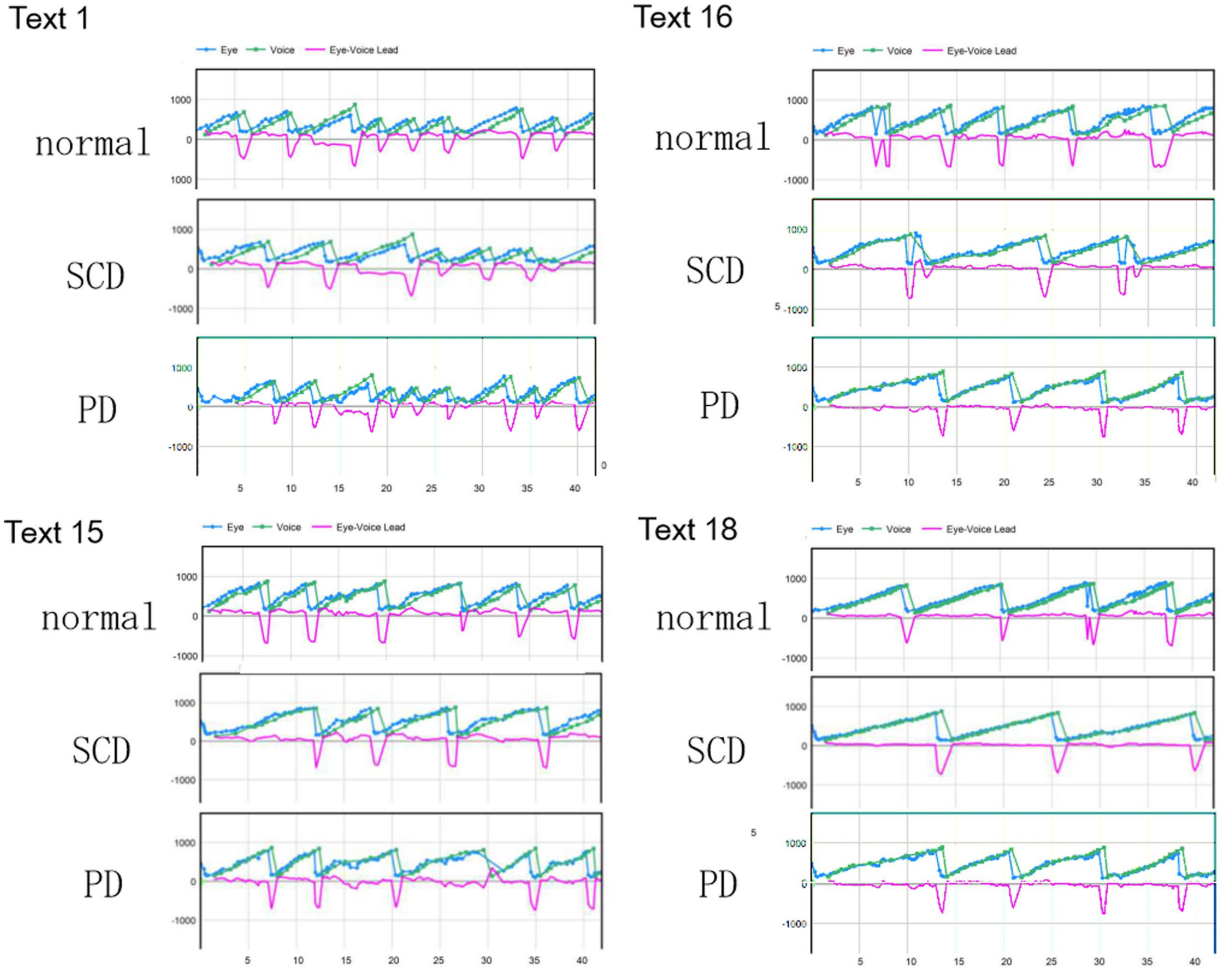
Typical examples of eye–voice coordination during reading aloud. The abscissa shows the time and the ordinate shows the gaze (blue curves) and read positions (green curves) of the text (x-coordinate of the monitor screen). Plots for each text (texts 1, 15, 16, and 21) are given separately for normal subjects (top row), SCD (middle row), and PD patients (bottom row). Note that the gaze position precedes the read (uttered position) except where line changes occur. The pink curve in each figure shows the spatial eye–voice lead (EVL), i.e., the distance by which the gaze position preceded the reading position. The pink curve in each figure shows the spatial EVL, i.e., the distance by which the gaze position preceded the read (uttered) position. Normal: normal subjects, PD: patients with Parkinson’s disease, SCD: patients with spinocerebellar degeneration.

The gaze position data were processed using EyeLink Data Viewer software (Data Viewer ver. 1.3.137., SR Research, Mississauga, Ontario, Canada). Based on the input from the microphone and the gaze data output from the Eyelink, two parallel datasets were generated: an eye movement dataset and a sound file in the wave format. Eye movement and audio input data were synchronized and put in the register in time using a custom program operating on Experiment builder that was obtained from the support page of SR research, which was provided by De Luca et al. (2013). The time relationship between audio recordings and gaze position (EVL) was analyzed offline using a custom-made program produced using Visual Studio 2015 (Microsoft, Redmond, Washington). The voice-line recording onset and offset was automatically overlaid on the timeline of the eye movements recording output (Figure 2). From the overlaid timelines of the eye movements recording and voice recordings, we analyzed for each moment, (1) Where the gaze was looking at, to place the x-coordinates of the gaze (gaze position), and (2) the read positions on the text (uttered voice position). Both data were then put together.

Saccade reports generated by the Data Viewer software provided data such as the saccade amplitude, x- and y-coordinates of the start and end positions of the saccade, and start and end times of saccades, whereas the fixation report contained the x- and y-coordinates of each fixation, the start and end time of the fixation, and the duration of individual fixation in each trial (each text reading).

Based on these, we calculated the following saccade parameters for each trial (text): the average number of saccades made per second (saccade frequency), mean amplitude of saccades, mean saccade duration, mean fixation duration, and proportion of regressive saccades made during reading (%). Gaze duration was defined as the sum of fixation and saccade duration.

By listening to the recording as the text was read aloud, the text position the subject was currently reading (reading or uttered position) was marked on the screenshot interface along the timeline interface of the program and the time was recorded simultaneously. Listening to the wave files, fixation positions were mapped onto letter positions over the screenshot of the three-line sentence to determine which graphemes in the passage were fixated.

Reading speed was defined as the average speed that the uttered position (x- and y-coordinates of the screen position in pixels) moved over the text as reading proceeded from left to right, excluding positions where there were line changes in the text. The EVL was the distance by which the eye moved ahead of the voice during reading aloud. More specifically, it refers to differences between fixated and uttered words, which is related to processing difficulty at a given point in time (when the gaze lagged behind the voice the eye-EVL was negative), as the subjects read each text.

Since the overall general level of spatial EVL was relatively stable for each text, even adjusting for different text sizes (see Results), we took the average EVL as an overall indicator for both onset and offset EVL. This parameter corresponds to spatial EVS in previous literature (Laubrock and Kliegl, 2015), as opposed to temporal EVS, which is frequently discussed in previous literature. Temporal EVL has been defined at both the onset of gaze in a certain word (onset EVL) and the offset of gaze from the word (offset EVL) (Silva et al., 2016). However, since there is no space between words in typical Japanese texts, except at explicit punctuations, we could not clearly define clear onset or offset EVLs. Instead, we took the average spatial EVL as an overall indicator for spatial EVL, since the overall general level of EVL was relatively stable for each text. In view of the relatively stable EVL for each text and the relatively stable reading speed for each text, we calculated the temporal EVL by dividing the former by the latter. Reading parameters, such as reading velocity and EVL, were expressed in letter values where appropriate.

The instantaneous location of the text gazed at by the eyes (gaze position), from the eye tracker appeared simultaneously on the interface timeline. In this way, the instantaneous gaze and reading positions (x- and y-coordinates) were marked consecutively at each moment over the screenshot as pixels on the screen. This information was integrated into a single plot, depicting the gaze and reading positions as a function of time (Figure 1). From this plot, the distance by which the gaze position led the reading position was calculated at each moment of the recording (EVL).

From the data, reading speed (letters/s) was calculated as the mean speed of reading the text in number of letters, excluding positions of the text where line changes took place. Although gaze movements actually comprise discrete movements (saccades) intervening with fixation periods, the overall speed of gaze scanning the text was also calculated, excluding where line changes occurred and where regressive eye movements occurred. This was defined as the product of saccade frequency and saccade amplitude multiplied by the proportion of non-regressive saccades, a parameter reflecting the speed of gaze scanning the text, excluding the contribution of regressive saccades.

To assess the degree to which parallel processing was taking place during reading, for the word gazed at and the word to be uttered, we calculated the ratio of gaze duration to temporal EVL according to Silva et al. (2016). This ratio reflects the higher weight of different processing stages (gaze-dependent vs. gaze-independent) within the onset EVS, with a higher ratio associated with more weight in the gaze-dependent process (see Discussion).

We also measured saccade parameters during the reading-aloud task: the number of saccades per unit time, the mean amplitude of saccades, such as the mean duration of fixation (ms), mean saccade duration (ms), mean saccade amplitude (deg), number of saccades made per unit time (/s), proportion of regression (regressive saccades among the total number of saccades [%]), and finally EVL (in pixels or number of letters). EVL (in letters) was defined as the amount of time by which gaze preceded the voice and was compared among the subject groups (PD, SCD patients, and normal subjects).

### Statistical analysis

Statistical analyses were performed using a commercial software (version 19.0; SPSS Inc., Chicago, IL). To analyze the saccade and reading parameters (saccade amplitude, saccade frequency, frequency of regression, EVL), repeated measures analysis of variance (ANOVA) was conducted with a within-subject factor: subject group (3 levels, PD, SCD patients, and normal subjects) and a between-subject factor: the read text (22 levels), where appropriate. The significance criteria were set at a *p*-value of less than 0.05. Contingent on the significance of analyzed effects, *post hoc* analyses were also conducted, using the Bonferroni/Dunn’s correction for multiple comparisons. We also analyzed the correlation between disease stage and the parameters within the two patient groups using the Spearman’s rank correlation in both SCD and PD groups.

## Results

### Eye–hand coordination during reading

Figure 1 shows typical examples of eye–voice coordination during reading, in which the abscissa gives the time and the ordinate the gaze (blue curves) read positions (green curves) of the text (x-coordinate of the monitor screen). SCD patients exhibited a slower reading speed compared with normal subjects and PD patients, as reflected in the flatter slope of the plot depicting the reading positions as a function of time. During the course of reading, gaze was sometimes directed backwards (from right to left), which was termed *regression* or *regressive saccades*. Although slightly more frequent in SCD patients, regression was observed in all subject groups.

In all subject groups, the similar slope of curves for the gaze (blue curves) and voice (green curves) indicated that the overall reading speed and gaze movements were similar, although they showed some variability. The pink curve in each figure shows the spatial EVL, that is, the distance by which the gaze position preceded the position of the uttered letter at each moment. For all groups of subjects, on average, the gaze led the uttered letter almost constantly by 2.9 to 3.2 letters throughout the text reading (Table 3), except at locations where line changes occurred.

**Table 3:** Reading and saccade parameters during reading.

Parameter	Normal	PD	SCD
Reading vel (letter/s)	4.71 ± 2.38	4.79 ± 3.13	3.53 ± 1.81
Saccade amplitude (deg)	3.88 ± 1.62	3.38 ± 1.28	3.56 ± 1.14
Saccade frequency (/s)	5.06 ± 1.21	2.84 ± 0.51	3.80 ± 0.71
Fixation duration (ms)	236.8 ± 70.4	304.0 ± 66.2	293.3 ± 60.9
Saccade duration (ms)	74.3 ± 38.9	58.9 ± 35.1	57.3 ± 30.8
Gaze duration (ms)	309.5 ± 61.1	356.8 ± 60.1	365.0 ± 69.9
Scanning speed (letter/s)	12.55 ± 5.42	9.64 ± 4.26	10.81 ± 4.52
Regression (%)	22.9 ± 6.6	23.6 ± 6.7	27.1 ± 8.5
Eye voice lead (EVL) (letter)	2.95 ± 1.17	2.95 ± 1.51	3.21 ± 1.35
Eye voice lead (EVL) (ms)	664 ± 242	683 ± 493	1041 ± 404

Values represent mean ± standard deviation.

Except where line changes occurred, EVL was relatively stable during text reading, as shown by the slope of the correlation between time and EVL. However, after closer inspection, there were occasions on which the gaze position temporarily led the reading position largely, especially at the beginning of the texts in normal subjects and SCD patients, but later decreasing to zero. In contrast, the gaze of PD would occasionally lag behind the reading position, resulting in slightly negative EVL.

For all subject groups, the reading speed as well as the EVL reliably decreased as the readability of the text decreased and texts were more difficult to read or could be read only at slower speed. For increasing number of texts (as shown in Figure 2), text readability decreased, that is, the text became more difficult to read speedily. Although reading speed expressed in pixels over the monitor screen was also slower as the individual letter size became smaller, reading speed was similar across texts with similar levels of text readability when expressed in terms of the number of letters. Thus, in the following analyses, the reading speed was expressed in terms of the letters in each text (letters/s).

### Parameters of reading in normal subjects and neurological patients

Quantitative analyses corroborated the above visual inspection (reading and saccade parameters summarized in Table 3). For statistical assessment, we separately analyzed typical Japanese texts without spaces [text between words (texts 1–19 in Table 2)] and a word list arranged with spaces (texts 20–22 in Table 2; statistical results summarized in Tables 4, 5, for analyses of SCD patients restricting to MSAC patients, Tables 4_2, 5_2, respectively).

**Table 4:** Analysis of variance results for reading and saccade parameters with disease stage (texts 1–19).

Variable	Group		Text		Group X Text	
	*F*_(2,124)_	*p*	*F*_(21,1302)_	*p*	*F*_(42,2604)_	*p*
Reading velocity	*F* = 10.433	*p* = 0.0002*	*F* = 224.890	*p* < 0.0001**	*F* = 2.161	*p* = 0.0001*
Saccade amplitude	*F* = 1.674	*p* = 0.1964	*F* = 115.781	*p* < 0.0001**	*F* = 1.695	*p* = 0.0068*
Saccade frequency	*F* = 14.273	*p* < 0.0001**	*F* = 0.423	*p* = 0.6560	*F* = 1.069	*p* = 0.3755
Fixation duration	*F* = 0.327	*p =* 0.7222	*F* = 16.536	*p* < 0.0001**	*F* = 3.023	*p* < 0.0001**
Saccade duration	*F* = 1.343	*p =* 0.2691	*F* = 7.152	*p* < 0.0001**	*F* = 1.098	*p* = 0.3196
Gaze duration	*F* = 0.554	*p* = 0.5781	*F* = 12.876	*p* < 0.0001**	*F* = 2.649	*p* < 0.0001**
Regression	*F* = 3.531	*p* = 0.0341*	*F* = 13.697	*p* < 0.0001**	*F* = 2.537	*p* < 0.0001**
Scanning speed	*F* = 11.813	*p* < 0.0001**	*F* = 88.019	*p* < 0.0001**	*F* = 3.65	*p* < 0.0001**
Scanning – reading speed	*F* = 5.589	*p* = 0.0070	*F* = 29.196	*p* < 0.0001**	*F* = 2.167	*p* = 0.0001*
Eye voice lead (EVL)	*F* = 0.716	*p* = 0.4948	*F* = 51.891	*p* < 0.0001**	*F* = 0.995	*p* = 0.4785
Variability of EVL	*F* = 0.775	*p* = 0.4682	*F* = 1.130	*p* = 0.3176	*F* = 1.123	*p* = 0.2887
Gaze duration/temporal EVL	*F* = 4.834	*p* = 0.0135*	*F* = 3.978	*p* < 0.0001**	*F* = 1.138	*p* = 0.0810

*Significance at *p* < 0.05; **Significance at *p* < 0.0001.

**Table 4_2:** Analysis of variance results for reading and saccade parameters with disease stage (analysis of SCD patients restricted to MSAC patients, texts 1–19).

Variable	Group		Text		Group X Text	
	*F*_(2,110)_	*p*	*F*_(21,1155)_	*p*	*F*_(42,2310)_	*p*
Reading velocity	*F* = 8.924	*p* = 0.0007*	*F* = 215.495	*p* < 0.0001**	*F* = 1.680	*p* = 0.0084*
Saccade amplitude	*F* = 1.961	*p* = 0.1508	*F* = 82.309	*p* < 0.0001**	*F* = 1.648	*p* = 0.0101*
Saccade frequency	*F* = 11.876	*p* < 0.0001**	*F* = 10.579	*p* < 0.0001**	*F* = 1.672	*p* = 0.0084
Fixation duration	*F* = 0.144	*p =* 0.8659	*F* = 11.967	*p* < 0.0001**	*F* = 2.662	*p* < 0.0001**
Saccade duration	*F* = 1.185	*p =* 0.3137	*F* = 7.032	*p* < 0.0001**	*F* = 1.222	*p* = 0.1755
Gaze duration	*F* = 0.238	*p* = 0.7888	*F* = 9.120	*p* < 0.0001**	*F* = 2.302	*p* < 0.0001**
Regression	*F* = 2.276	*p* = 0.1131	*F* = 11.330	*p* < 0.0001**	*F* = 2.347	*p* < 0.0001**
Scanning speed	*F* = 13.324	*p* < 0.0001**	*F* = 65.461	*p* < 0.0001**	*F* = 3.529	*p* < 0.0001**
Scanning – reading speed	*F* = 5.472	*p* = 0.0083*	*F* = 9.443	*p* < 0.0001**	*F* = 2.247	*p* < 0.0001**
Eye voice lead (EVL)	*F* = 0.143	*p* = 0.8674	*F* = 37.813	*p* < 0.0001**	*F* = 1.070	*p* = 0.3619
Variability of EVL	*F* = 0.654	*p* = 0.5270	*F* = 0.7550	*p* = 0.7541	*F* = 0.899	*p* = 0.6395
Gaze duration/temporal EVL	*F* = 2.178	*p* = 0.1288	*F* = 3.038	*p* < 0.0001**	*F* = 0.840	*p* = 0.7342

*Significance at *p* < 0.05; **Significance at *p* < 0.0001.

**Table 5:** Analysis of variance results for reading and saccade parameters with disease stage (texts 20–22).

Parameter	Group		Text		Group X Text	
	*F*_(2,124)_	*p*	*F*_(21,1302)_	*p*	*F*_(42,2604)_	*p*
Reading velocity	*F* = 15.085	*p* < 0.0001**	*F* = 151.667	*p* < 0.0001**	*F* = 15.845	*p* < 0.0001**
Saccade amplitude	*F* = 1.417	*p* = 0.2504	*F* = 66.275	*p* < 0.0001**	*F* = 2.238	*p =* 0.0689
Saccade frequency	*F* = 12.190	*p* < 0.0001**	*F* = 14.518	*p* < 0.0001**	*F* = 1.465	*p* = 0.0389*
Fixation duration	*F* = 2.511	*p =* 0.0870	*F* = 6.372	*p* = 0.0023*	*F* = 1.005	*p* = 0.4079
Saccade duration	*F* = 0.356	*p =* 0.7018	*F* = 0.450	*p =* 0.6387	*F* = 0.555	*p* = 0.6960
Gaze duration	*F* = 2.539	*p* = 0.0875	*F* = 4.190	*p* = 0.0175*	*F* = 0.997	*p* = 0.4120
Regression	*F* = 2.961	*p* = 0.0595	F = 2.714	*p* = 0.0704	*F* = 1.258	*p* = 0.2906
Scanning speed	*F* = 8.879	*p* = 0.0004*	*F* = 53.479	*p* < 0.0001**	*F* = 1.874	*p* < 0.0001**
Scanning – reading speed	*F* = 31.521	*p* < 0.0001**	*F* = 53.177	*p* < 0.0001**	*F* = 14.154	*p* < 0.0001**
Eye–voice lead (EVL)	*F* = 1.891	*p* = 0.1607	*F* = 73.782	*p* < 0.0001**	*F* = 5.436	*p* = 0.0005
Variability of EVL	*F* = 0.797	*p* = 0.4572	*F* = 36.832	*p* < 0.0001**	*F* = 1.915	*p* = 0.1149
Gaze duration/temporal EVL	*F* = 3.323	*p* = 0.0434*	*F* = 52.716	*p* < 0.0001**	*F* = 6.252	*p* = 0.0001

*Significance at *p* < 0.05; **Significance at *p* < 0.0001.

**Table 5_2:** Analysis of variance results for reading and saccade parameters with disease stage (analysis of SCD patients restricted to MSAC patients, texts 20–22).

Parameter	Group		Text		Group X Text	
	*F*_(2,110)_	*p*	*F*_(2,110)_	*p*	*F*_(4,220)_	*p*
Reading velocity	*F* = 12.147	*p* < 0.0001**	*F* = 97.150	*p* < 0.0001**	*F* = 14.926	*p* < 0.0001**
Saccade amplitude	*F* = 1.782	*p* = 0.1781	*F* = 57.751	*p* < 0.0001**	*F* = 2.082	*p =* 0.0881
Saccade frequency	*F* = 13.139	*p* < 0.0001**	*F* = 0.564	*p* = 0.5705	*F* = 0.900	*p* = 0.4669
Fixation duration	*F* = 2.060	*p =* 0.1374	*F* = 4.872	*p* = 0.0094*	*F* = 0.540	*p* = 0.7065
Saccade duration	*F* = 0.360	*p =* 0.6991	*F* = 0.868	*p =* 0.9346	F = 0.868	*p* = 0.4859
Gaze duration	*F* = 2.029	*p* = 0.1416	*F* = 4.552	*p* = 0.0130*	*F* = 0.595	*p* = 0.6673
Regression	*F* = 1.644	*p* = 0.2027	*F* = 2.714	*p* = 0.0700	*F* = 1.306	*p* = 0.2723
Scanning speed	*F* = 9.204	*p* = 0.0004*	*F* = 33.485	*p* < 0.0001**	*F* = 2.057	*p =* 0.0917
Scanning - reading speed	*F* = 27.641	*p* < 0.0001**	*F* = 34.086	*p* < 0.0001**	*F* = 12.516	*p* < 0.0001**
Eye–voice lead (EVL)	*F* = 3.296	*p* = 0.0454	*F* = 42.454	*p* < 0.0001**	*F* = 5.563	*p* = 0.0004*
Variability of EVL	*F* = 0.724	*p* = 0.4914	*F* = 21.063	*p* < 0.0001**	*F* = 1.859	*p* = 0.1263
Gaze duration/temporal EVL	*F* = 2.116	*p* = 0.1314	*F* = 34.128	*p* < 0.0001**	*F* = 5.735	*p* = 0.0003

*Significance at *p* < 0.05; **Significance at *p* < 0.0001.

**Figure 3:**
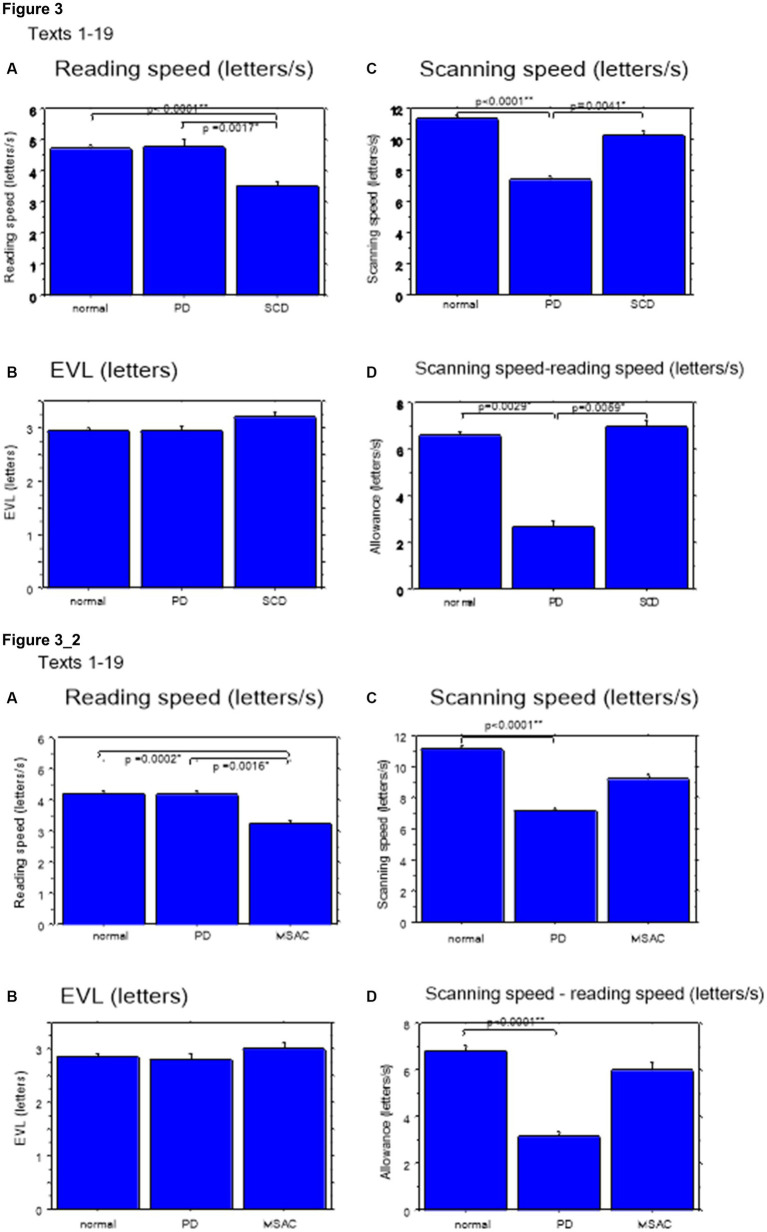
Reading parameters for reading texts in normal subjects, PD and SCD patients (text 1–19). **(A)** Reading speed (letters/s), **(B)** Eve–voice lead (EVL) (letters), **(C)** Scanning speed (letters/s), **(D)** Scanning speed-reading speed (letters/s). Normal: normal subjects, PD: patients with Parkinson’s disease, SCD: patients with spinocerebellar degeneration. Error bars show standard errors. Asterisks indicate significant difference at *p < 0.05, or **p < 0.0001. **3_2** (lower four figures): Reading parameters for reading texts in normal subjects, PD and MSAC patients (text 1–19). Similar figures with conventions as in Figure 3 when the analysis of SCD patients were restricted to MSAC patients. MSAC, multiple system atrophy cerebellar-type.

**Figure 4:**
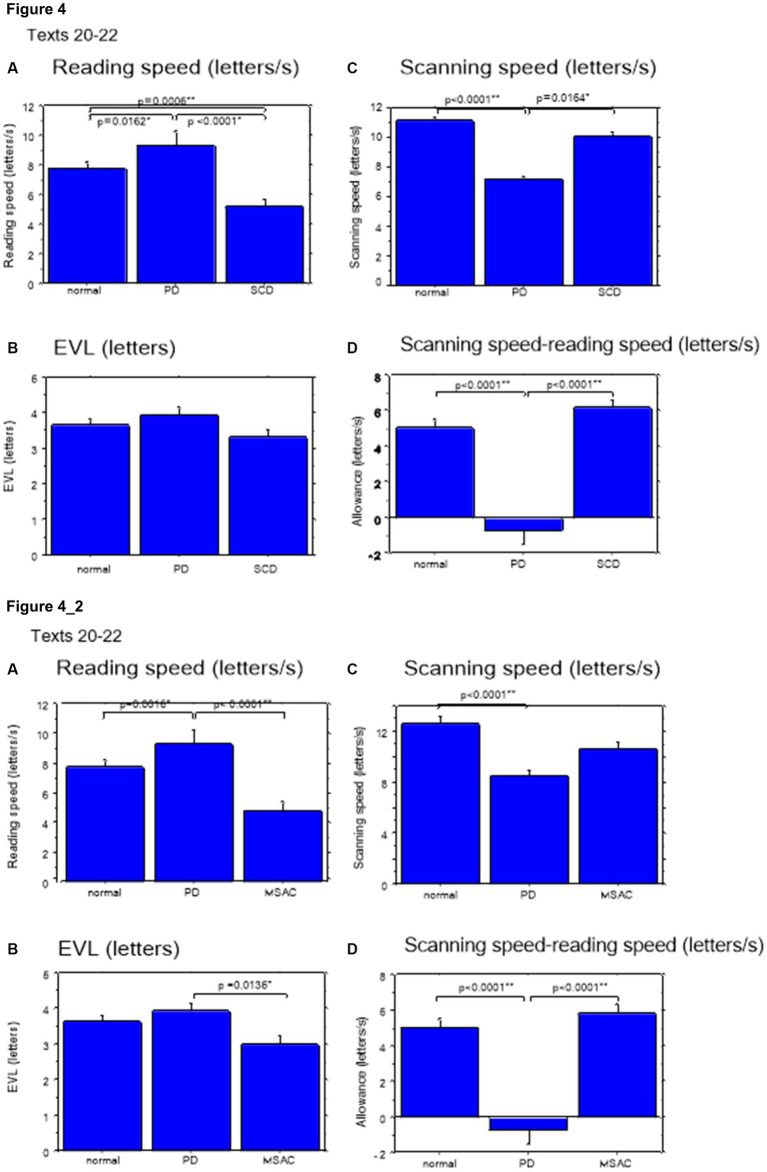
Reading parameters in normal subjects, PD and SCD patients (text 20–22). Similar figures with conventions as in Figure 3. **(A)** Reading speed (letters/s), **(B)** Eve–voice lead (EVL) (letters), **(C)** Scanning speed (letters/s), **(D)** Scanning speed-reading speed (letters/s). **4_2** (lower four figures): Reading parameters for reading texts in normal subjects, PD and MSAC patients (text 20–22). Similar figures with conventions as in Figure 4 when the analysis of SCD patients were restricted to MSAC patients. MSAC, multiple system atrophy cerebellar-type.

Excluding places where line changes occurred, reading speed was significantly slower in SCD patients compared with normal subjects and even PD patients (Figure 3A, *post hoc* analysis: normal vs. SCD *p* < 0.0001, PD vs. SCD *p* = 0.0017). This trend persisted even when we restricted the analysis of SCD patients to MSAC patients (Figure 3_2A, *post hoc* analysis: normal vs. SCD *p* = 0.0002, PD vs. SCD *p* = 0.0016). In PD, reading speed was comparable to normal subjects. The general trend was similar for the three texts that consisted of an arranged list of words or phonograms with spaces in between (text 20–22), although the reading speed was slightly faster for PD, followed by normal subjects and then SCD patients (Figure 4A; *post hoc* analysis: normal vs. PD *p* = 0.0162, normal vs. SCD *p* = 0.0006, PD vs. SCD *p* < 0.0001). This trend also held true when we restricted our analysis of SCD patients to MSAC patients (Figure 4_2A, *post hoc* analysis: normal vs. SCD *p* = 0.0018, PD vs. SCD *p* < 0.0001).

Reading speed correlated negatively with disease stage in SCD patients (correlation results with disease stage are presented in Table 6 for texts 1–19, and Table 7 for texts 20–22). This was true even restricting the analysis of SCA patients to MSAC patients (rightmost columns of Tables 6, 7). In PD patients, there was a slight negative correlation between disease stage (UPDRS motor score) and average reading speed across texts, but this correlation failed to reach significance.

**Table 6:** Statistical results for the correlation analyses between saccade parameters and disease stage (texts 1–19).

Parameters	PD		SCD		MSAC	
	*r*	*p*	*r*	*p*	*r*	*p*
Reading speed	*r* = −0.334	*p* = 0.1648	*r* = −0.571	*p* = 0.0246*	*r* = 0.775	*p* = 0.0211*
Saccade amplitude	*r* = −0.333	*p* = 0.1659	*r* = −0.484	*p* = 0.0670	*r* = 0.135	*p* = 0.7613
Saccade duration	*r* = −0.393	*p* = 0.0965	*r* = 0.144	*p* = 0.6161	*r* = 0.019	*p* = 0.9660
Fixation duration	*r* = 0.347	*p* = 0.1479	*r* = 0.495	*p* = 0.0599	*r* = 0.225	*p* = 0.6095
Gaze duration	*r* = −0.180	*p* = 0.4658	*r* = 0.597	*p* = 0.0170*	*r* = 0.234	*p* = 0.5934
Saccade frequency	*r* = −0.258	*p* = 0.2901	*r* = −0.425	*p* = 0.1161	*r* = 0.038	*p* = 0.9323
Scanning speed (saccade amplitude × saccade frequency)	*r* = −0.416	*p* = 0.0465	*r* = −0.649	*p* = 0.0073*	*r* = 0.178	*p* = 0.6872
Frequency of regression	*r* = 0.469	*p* = 0.0418	*r* = −0.241	*p* = 0.3951	*r* = 0.577	*p* = 0.1413
Eye–voice lead (EVL)	*r* = 0.208	*p* = 0.3993	*r* = −0.208	*p* = 0.4636	*r* = 0.078	*p* = 0.8618
Eye–voice lead (temporal EVL)	*r* = 0.418	*p* = 0.0847	*r* = 0.092	*p* = 0.7494	*r* = 0.545	*p* = 0.1344
Gaze duration/temporal EVL	*r* = −0.363	*p* = 0.1284	*r* = −0.024	*p* = 0.9343	*r* = −0.662	*p* = 0.0750

*Significance at *p* < 0.05.

**Table 7:** Statistical results for the correlation analyses between saccade parameters and disease stage (texts 20–22).

Parameters	PD		SCD		MSAC	
	*r*	*p*	*r*	*p*	*r*	*p*
Reading speed	*r* = −0.274	*p* = 0.2757	*r* = −0.593	*p* = 0.0236*	*r* = −0.317	*p* = 0.4636
Saccade amplitude	*r* = −0.416	*p* = 0.0860	*r* = −0.352	*p* = 0.2232	*r* = 0.184	*p* = 0.6767
Saccade duration	*r* = −0.067	*p* = 0.7953	*r* = 0.344	*p* = 0.2349	*r* = 0.252	*p* = 0.5650
Fixation duration	*r* = −0.192	*p* = 0.4508	*r* = −0.057	*p* = 0.8511	*r* = 0.320	*p* = 0.4584
Gaze duration	*r* = −0.132	*p* = 0.6066	*r* = 0.153	*p* = 0.6083	*r* = 0.362	*p* = 0.3966
Saccade frequency	*r* = 0.010	*p* = 0.9684	*r* = −0.339	*p* = 0.2418	*r* = −0.024	*p* = 0.9581
Scanning speed (saccade amplitude × saccade frequency)	*r* = −0.340	*p* = 0.1696	*r* = −0.456	*p* = 0.1025	*r* = 0.170	*p* = 0.7009
Frequency of regression	*r* = 0.357	*p* = 0.1476	*r* = −0.241	*p* = 0.3951	*r* = 0.623	*p* = 0.1028
Eye–voice lead (spatial EVL)	*r* = 0.131	*p* = 0.6101	*r* = −0.606	*p* = 0.0199*	*r* = −0.368	*p* = 0.3886
Eye–voice lead (temporal EVL)	*r* = 0.404	*p* = 0.0967	*r* = −0.110	*p* = 0.7028	*r* = −0.277	*p* = 0.4852
Gaze duration/temporal EVL	*r* = −0.422	*p* = 0.0718	*r* = 0.069	*p* = 0.8183	*r* = 0.105	*p* = 0.8138

*Significance at *p* < 0.05.

### Saccade parameters during reading in normal subjects and neurological patients

**Figure 5:**
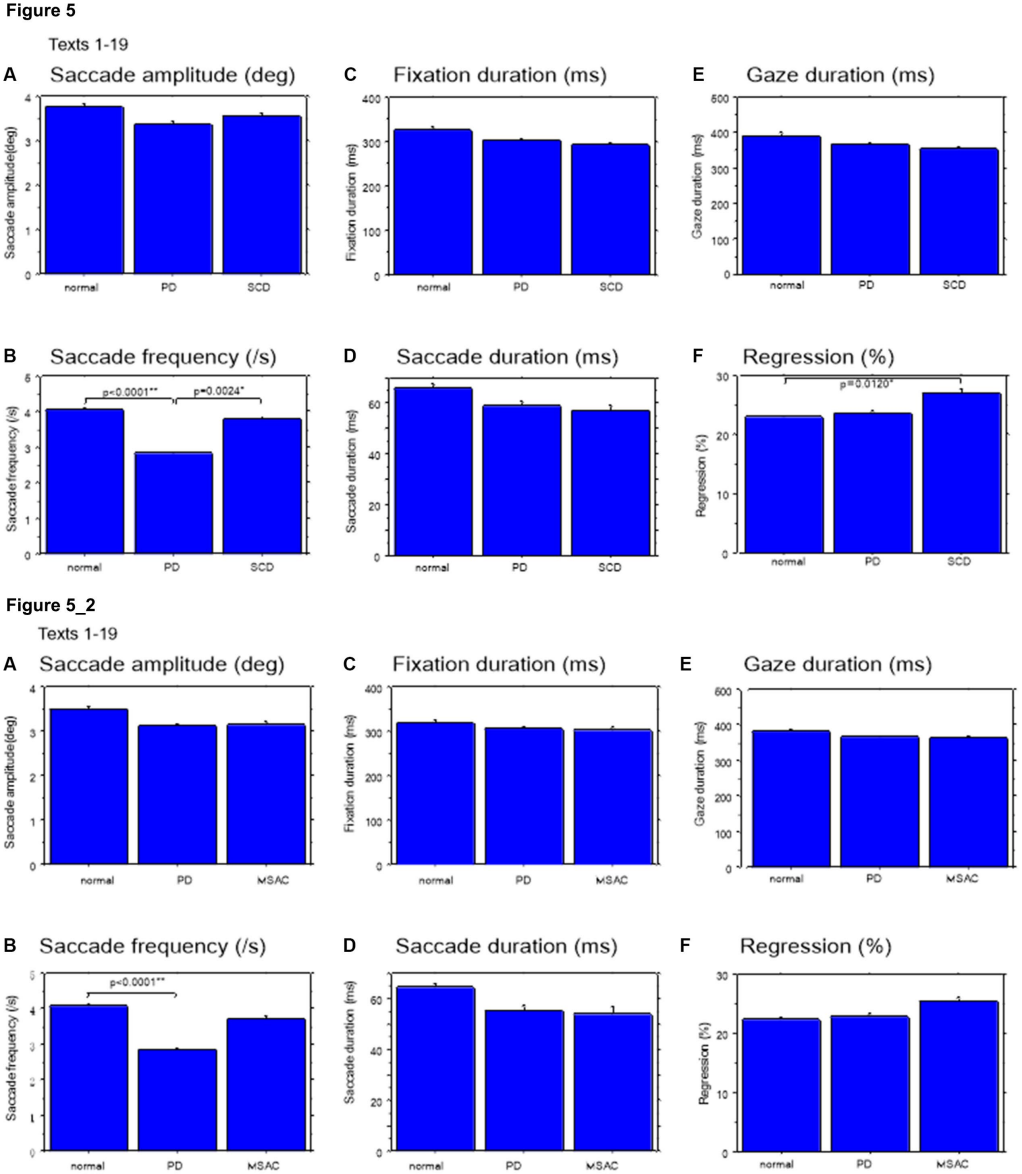
Saccade parameters during reading in normal subjects, PD and SCD patients (text 1–19). **(A)** Saccade amplitude (deg), **(B)** Saccade frequency (/s), **(C)** Fixation duration (ms), **(D)** Saccade duration (ms), **(E)** Gaze duration (ms), **(F)** Proportion of regressive saccades (%). Normal: normal subjects, PD: patients with Parkinson’s disease, SCD: patients with spinocerebellar degeneration. Error bars show standard errors. Asterisks indicate significant difference at *p < 0.05. **5_2**: (lower two rows) Saccade parameters during reading in normal subjects, PD and MSAC patients (text 1–19). Similar figures with conventions as in Figure 5 when the analysis of SCD patients were restricted to MSAC patients. MSAC, multiple system atrophy cerebellar-type.

**Figure 6:**
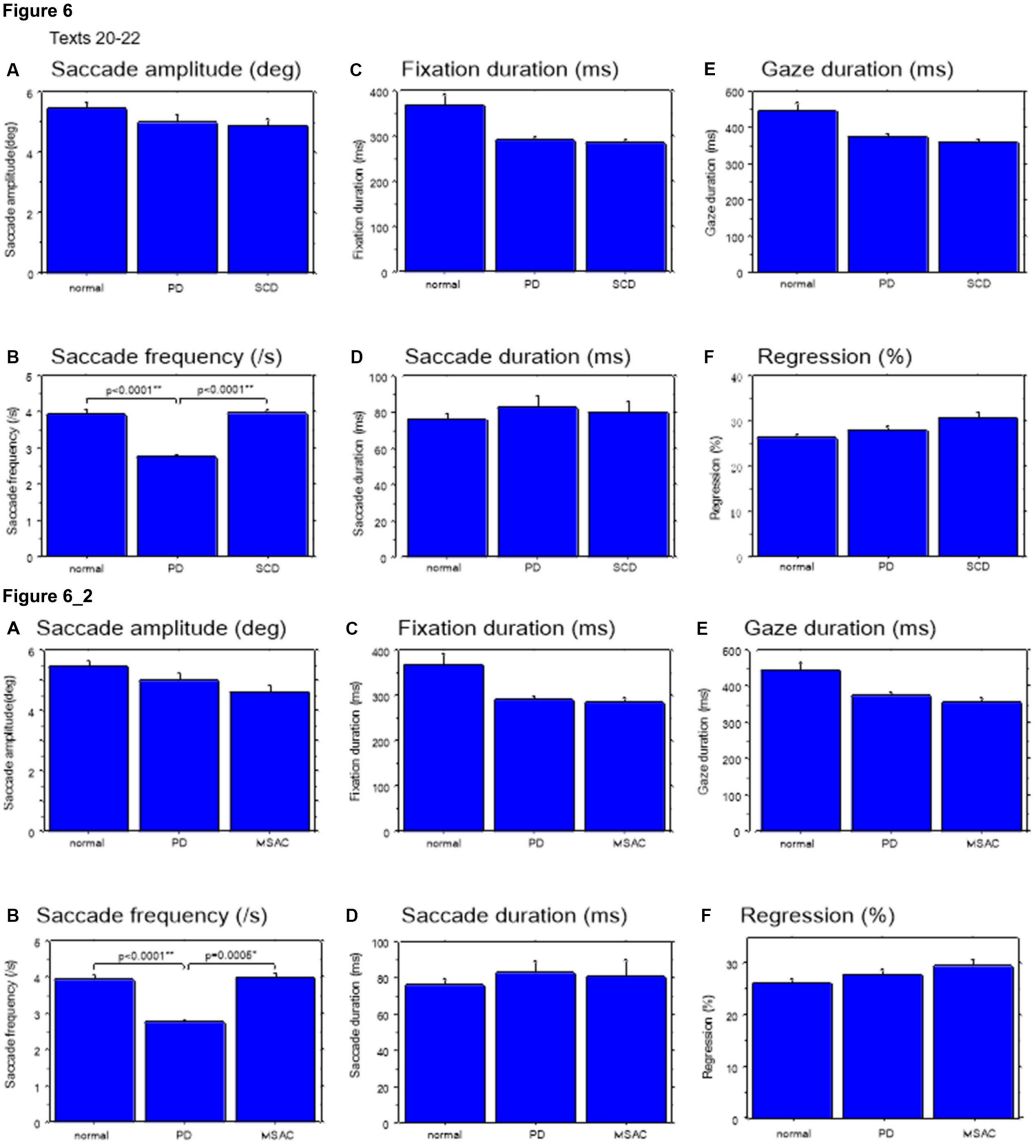
Saccade parameters during reading in normal subjects, PD and SCD patients (texts 20–22). Similar figures with conventions as in Figure 4. **(A)** Saccade amplitude (deg), **(B)** Saccade frequency (/s), **(C)** Fixation duration (ms), **(D)** Saccade duration (ms), **(E)** Gaze duration (ms), **(F)** Proportion of regressive saccades (%). Error bars show standard errors. Asterisks indicate significant difference at *p < 0.05. **6_2** (lower two rows): Saccade parameters during reading in normal subjects, PD and MSAC patients (text 20–22). Similar figures with conventions as in Figure 6 when the analysis of SCD patients were restricted to MSAC patients. MSAC, multiple system atrophy cerebellar-type.

We looked at the saccade parameters of gaze movements during oral reading and compared them among the three subject groups (Tables 4, 5). The mean saccade amplitude was slightly reduced in SCD and PD patients compared to normal subjects, but these differences did not reach significance (Figures 5A, 6A; restricting the analysis of SCD patients to MSAC patients Figures 5_2A, 6_2A). In contrast, saccade frequency was significantly lower in PD patients than in both normal subjects and SCD patients (Figures 5B, 6B; *post hoc* analysis: texts 1–19: normal vs. PD *p* < 0.0001, PD vs. SCD *p* = 0.00241; texts 20–22: normal vs. PD *p* < 0.0001, PD vs. SCD *p* < 0.0001; restricting the analysis of SCD patients to MSAC patients Figures 5_2B, 6_2B, normal vs. MSAC *p* = 0.0018, PD vs. MSAC *p* < 0.0001). Saccade frequency did not correlate significantly with disease stage in either PD and SCD patients for either texts 1–19 (Table 6) or texts 20–22 (Table 7). This held even restricting the analysis of SCA patients to MSAC patients (rightmost columns of Tables 6, 7).

In both SCD and PD patients, the fixation duration and saccade duration, though slightly shorter, were not significantly different from those of normal subjects (Tables 4, 5 and Figures 5C,D, 6C,D; restricting the analysis of SCD patients to MSAC patients Tables 4_2, 5_2, Figures 5_2C,D, 6_2C,D). Fixation gaze duration showed a significant negative correlation with disease stage only for texts 1–19 in SCD patients but not PD patients (Tables 6, 7). The lack of correlation held when restricting the analysis of SCD patients to MSAC patients. In SCD and PD patients, gaze duration was slightly but not significantly shorter than in normal subjects (Figures 5E, 6E; restricting the analysis of SCD patients to MSAC patients Figures 5_2E, 6_2E). Gaze durations showed a significant negative correlation with disease stage only for texts 1–19 in SCD patients but not PD patients. This significant correlation was lost when the analysis of SCD patients were restricted to MSAC patients.

For all subject groups, the position of gaze over the text led the uttered position of the text by approximately 3 letters on average (EVL, Table 3), consistent with the notion that the text was first scanned by the gaze and the visual input was transformed into vocal output (voice). EVL was statistically comparable among the three subject groups (Tables 4, 5 and Figures 3B, 4B; restricting the analysis of SCD patients to MSAC patients Tables 4_2, 5_2, Figures 3_2B, 4_2B). EVL, both spatial and temporal, did not correlate significantly with disease stage in either SCD or PD patents for any of the texts, except for texts 20–22 in SCD patients (Tables 6, 7). This significant correlation did not persist when the analysis of SCD patients were restricted to MSAC patients (right most columns in Tables 6, 7).

Except where line changes occurred, EVL was relatively stable during text reading. To assess the group differences, we studied the correlation between time and EVL. The slope of this correlation was not significantly different from 0 (slope: normal −0.26 ± 2.78 pixel/s, SCD −0.41 ± 1.8 pixel/s, PD −0.10 ± 0.80 pixel/s) and there were no differences in the slopes and intercept among subject groups (slope: effect of group: *F*(2,124) = 0.252, *p* = 0.7788; effect of text: *F*(21,1302) = 3.271, *p* < 0.0001, subject group X text: *F*(42,2604) = 1.183, *p* = 0.2074, intercept: effect of group: *F*(2,124) = 0.060, *p* = 0.9416; effect of text: *F*(21,1302) = 4.696, *p* < 0.0001, subject group X text: *F*(42,2604) = 1.558, *p* = 0.0150). The variability of EVL (standard deviation of EVL) across time was also similar for all groups across all texts, reflecting the overall similar EVL (*p* > 0.05) (not showed in Tables).

Gaze scanning the text sometimes involved regressive movements from right to left instead of proceeding progressively from left to right. Overall, the frequency of regression was significantly higher in SCD patients compared with normal subjects and PD patients for texts 1–19, but not for texts 20–22 (Tables 4, 5 and Figures 5F, 6F; *post hoc* analysis: texts 1–19: normal vs. SCD *p* = 0.0120; restricting the analysis to MSA patients Tables 4_2, 5_2; Figures 5_2F, 6_2F). The frequency of regression did not show significant correlation with disease stage or UPDRS motor score in either SCD or PD patients (Tables 4, 5).

These regressive saccades may impair rather than help advance processing of the text ahead. Thus, we considered the product of saccade amplitude multiplied by the frequency of saccades per unit time as an index of the potential speed of gaze scanning the text (hereafter termed “scanning speed”). This measure would approximately reflect the overall slope of the blue curves in Figure 1, excluding regressions. The scanning speed was reduced in PD patients relative to normal subjects and SCD patients (Figures 3C, 4C, *post hoc* analysis: texts 1–19 normal vs. PD *p* < 0.0001, normal vs. SCD *p* = 0.2342, SCD vs. PD *p* = 0.0041; texts 20–22 normal vs. PD *p* < 0.0001, normal vs. SCD *p* = 0.2227, SCD vs. PD *p* = 0.0164; restricting the analysis to MSA patients Figures 3_2C, 4_2C). Scanning speed significantly decreased progressively with disease stage in SCD patients for texts 1–19, also showing a tendency to decrease with UPDRS motor score in PD patients not reaching statistical significance (Tables 6, 7). This significant correlation did not persist when the analysis of SCD patients were restricted to MSAC patients (right most columns in Tables 6, 7).

How much the gaze position scanning the text could potentially precede the uttered text position was calculated by subtracting reading speed from scanning speed, that is, the allowance for the gaze to precede the uttered word position. This measure was reduced in PD patients relative to normal subjects and SCD patients (Figures 3D, 4D; *post hoc* analysis: text 1–19 normal vs. PD *p* = 0.0029, normal vs. SCD *p* = 0.8512, SCD vs. PD *p* = 0.0059; text 1–20-22 normal vs. PD *p* < 0.0001, normal vs. SCD *p* = 0.2447, SCD vs. PD *p* < 0.0001). This significant difference was seen also when the analysis of SCD patients were restricted to MSAC patients (Figures 3_2D, 4_2D). This implied that the potential scanning position of gaze led the uttered voice position in SCD patients and normal subjects more evidently than in PD patients.

### Reading and scanning speeds as a function of text readability

**Figure 7:**
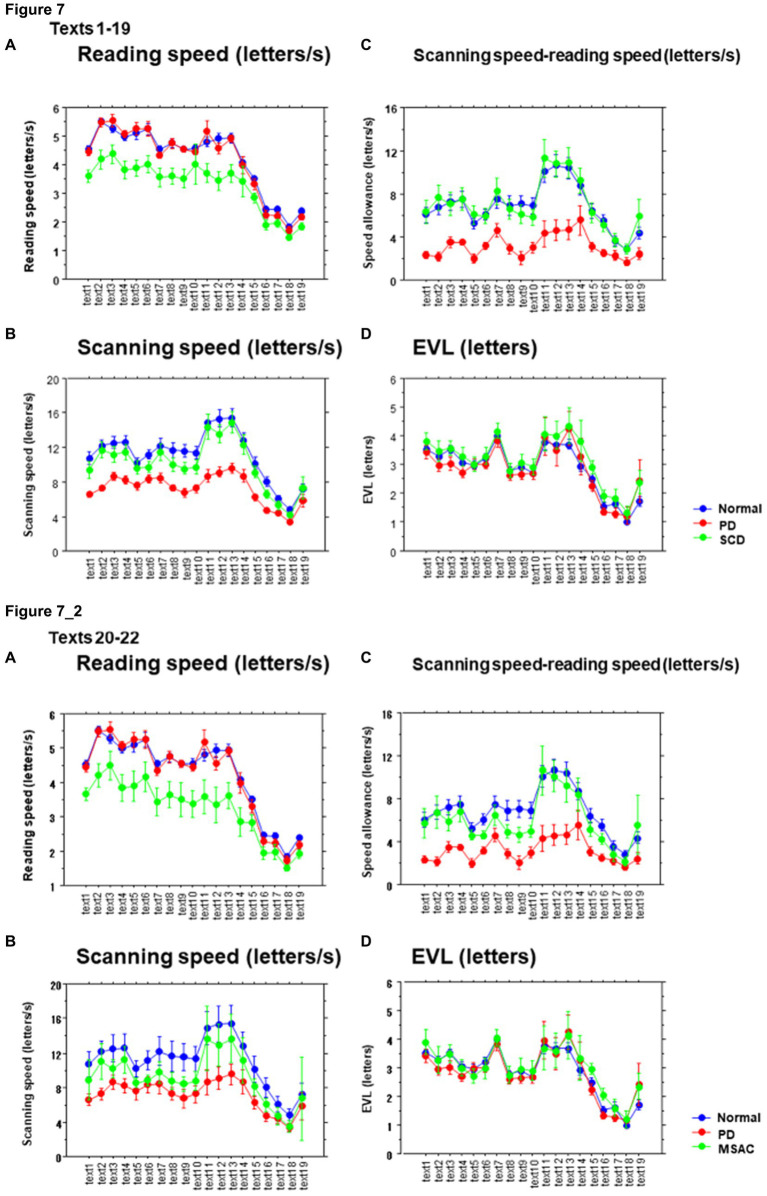
Reading parameters as a function of text readability (texts 1–19). **(A)** Reading speed (letters/s), **(B)** Scanning speed (letters/s), **(C)** Scanning speed-reading speed (letters/s), **(D)** Eye–voice lead (EVL, letters). Normal: normal subjects; PD: patients with Parkinson’s disease; SCD: patients with spinocerebellar degeneration. Text readability decreases from left to right along the ordinate. Error bars show standard errors. **7_2** (lower four figures): Reading parameters as a function of text readability (text 1–19). Similar figures with conventions as in Figure 7 when the analysis of SCD patients were restricted to MSAC patients. MSAC, multiple system atrophy cerebellar-type.

**Figure 8:**
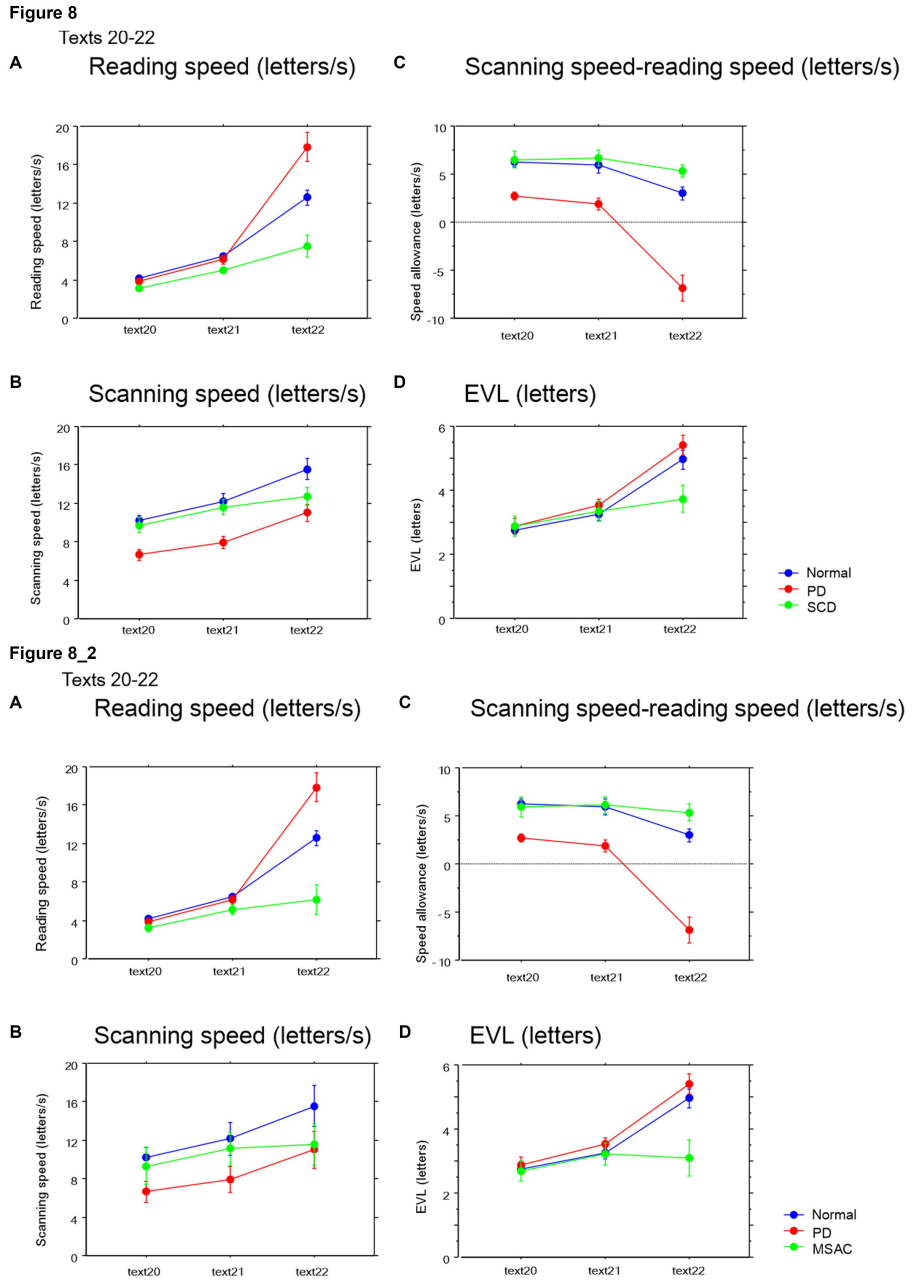
Reading parameters as a function of text readability (texts 20–22). Similar figures with conventions as in Figure 6. **(A)** Reading velocity (letters/s), **(B)** Scanning speed (letters/s), **(C)** Scanning speed-reading speed (letters), **(D)** Eye–voice lead (EVL, letters). **8_2** (lower four figures): Reading parameters as a function of text readability (text 20–22). Similar figures with conventions as in Figure 8 when the analysis of SCD patients were restricted to MSAC patients. MSAC, multiple system atrophy cerebellar-type.

We studied how the reading and saccade parameters changed with varying text readability (Tables 4, 5; restricting the analysis to MSA patients Tables 4_2, 5_2). Reading speed, as expected, decreased significantly with decreasing text readability (text becoming difficult to read) (Figures 7A, 8A; restricting the analysis to MSA patients Figures 7_2A, 8_2A). There was a significant interaction between subject group and text, reflecting the finding that, compared to PD patients and normal subjects, the reading speed of SCD patients was slower, especially for texts that were relatively easy-to-read and could be read faster (Tables 4, 5; restricting the analysis to MSA patients Tables 4_2, 5_2). In contrast, reading speed of PD patients was comparable to normal subjects for all texts (*p* = 0.6407), except for text 22, a Japanese syllabary.

**Figure 9:**
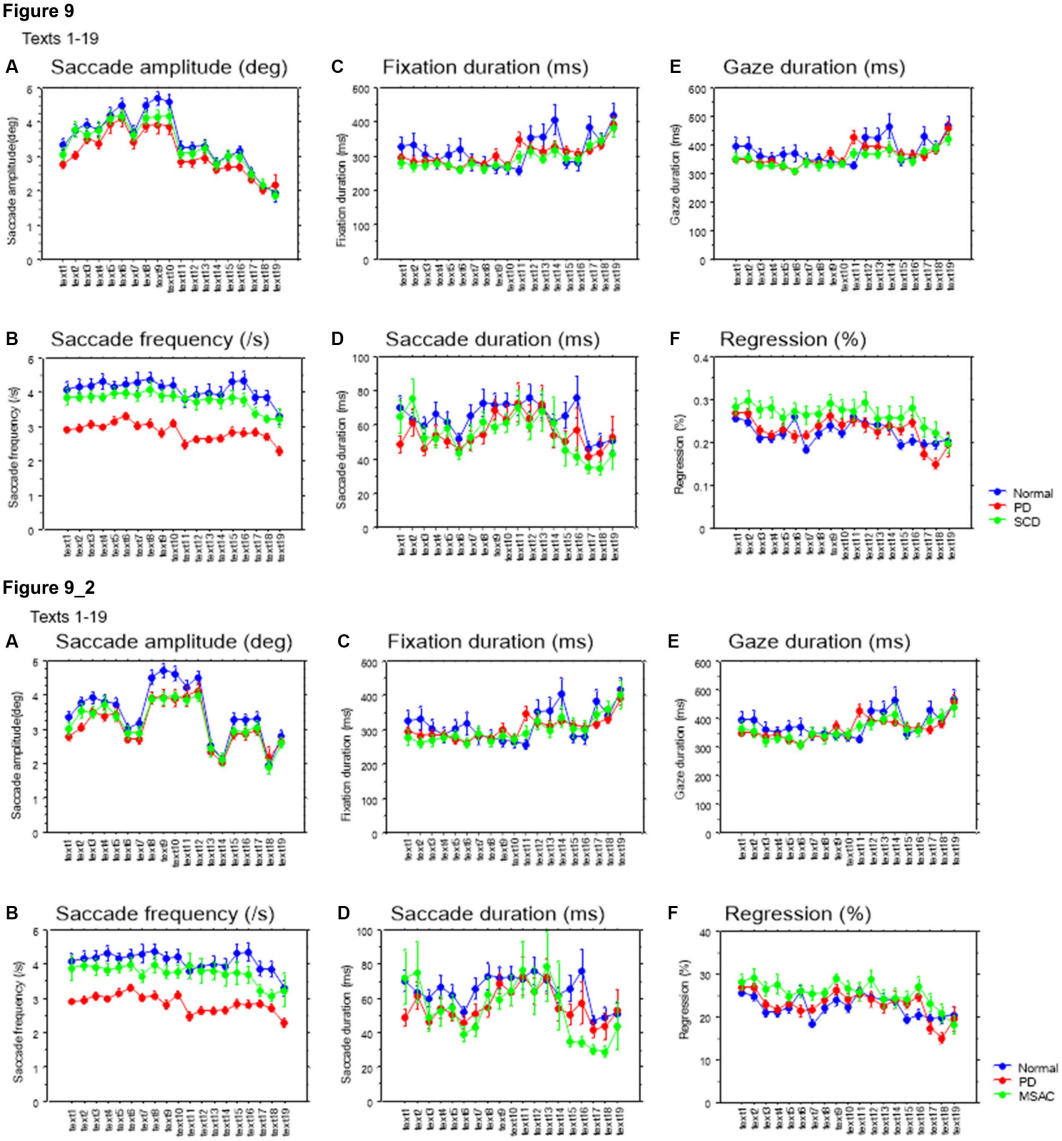
Saccade parameters as a function of text readability (texts 1–19). **(A)** Saccade amplitude (deg), **(B)** Saccade frequency (/s), **(C)** Fixation duration (ms), **(D)** Saccade duration (ms), **(E)** Gaze duration (ms), **(F)** Proportion of regressive saccades (%). Normal: normal subjects; PD: patients with Parkinson’s disease; SCD: patients with spinocerebellar degeneration. Text readability decreases from left to right along the ordinate. Error bars show standard errors. **9_2** (lower row): Saccade parameters as a function of text readability (text 1–19). Similar figures with conventions as in Figure 9 when the analysis of SCD patients were restricted to MSAC patients. MSAC, multiple system atrophy cerebellar-type.

**Figure 10:**
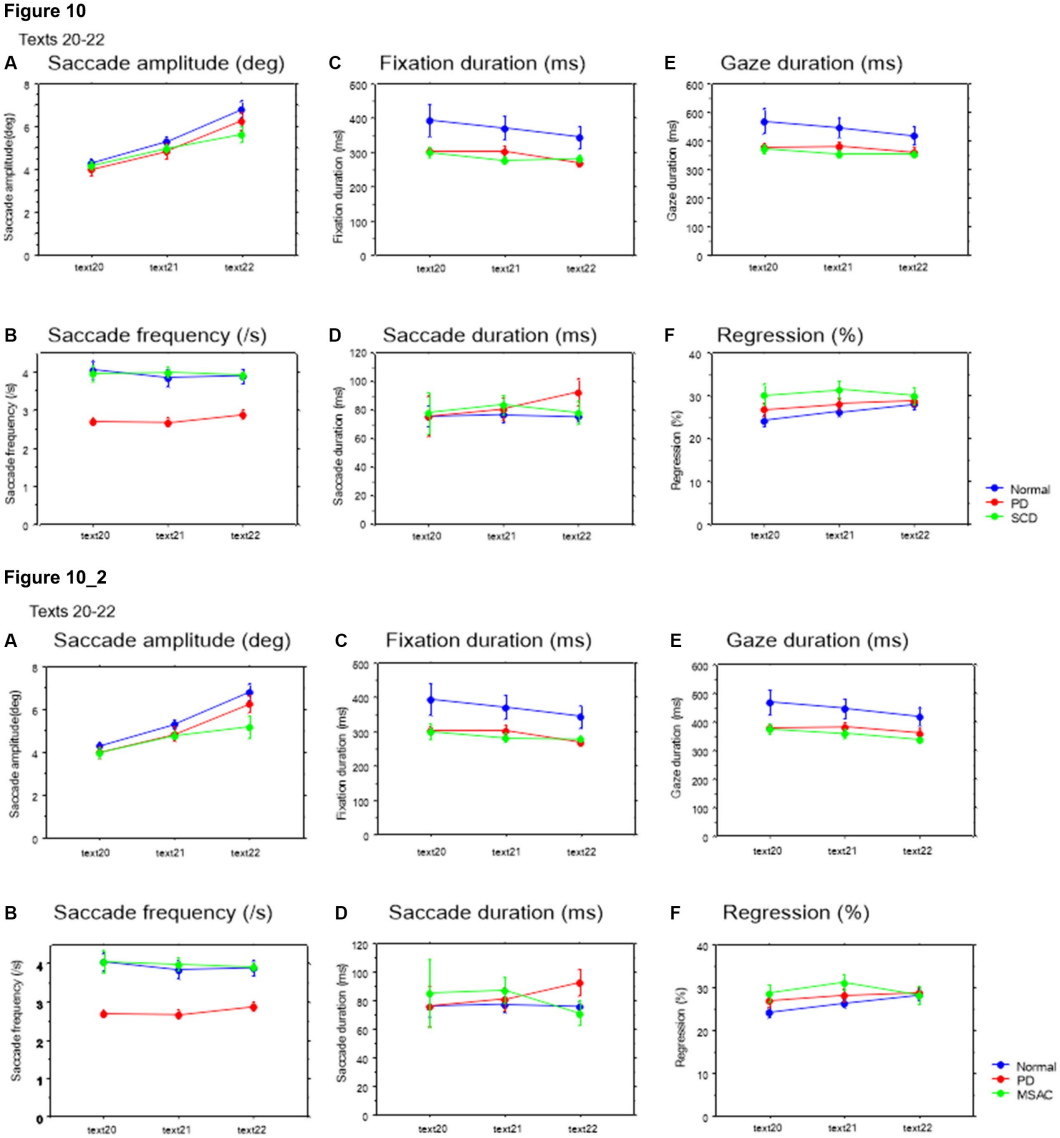
Saccade parameters as a function of text readability (texts 20–22). Similar figures with conventions as in Figure 8. **(A)** Saccade amplitude (deg), **(B)** Saccade frequency (/s), **(C)** Fixation duration (ms), **(D)** Saccade duration (ms), **(E)** Gaze duration (ms), **(F)** Proportion of regressive saccades (%). **10_2** (lower row): Saccade parameters as a function of text readability (text 20–22). Similar figures with conventions as in Figure 10 when the analysis of SCD patients were restricted to MSAC patients. MSAC, multiple system atrophy cerebellar-type.

Saccade amplitude reliably decreased as the text became difficult to read, but was statistically comparable among the three groups, and the interaction between subject group and text readability did not reach significance (Figures 9A, 10A; Tables 4, 5; restricting the SCD analysis to MSA patients Figures 9_2A, 10_2A; Tables 4_2, 5_2). Saccade frequency also significantly decreased for text 20–22 as text became difficult to read (text readability decreased; Figures 9B, 10B; restricting the analysis to MSA patients Figures 9_2B, 10_2B), and was consistently smaller in PD patients than in the other two groups across all text readability. Scanning speed decreased with increasing text readability, and was reduced in PD and SCD patients relative to normal subjects (Figures 7B, 8B; restricting the SCD analysis to MSA patients Figures 7_2B, 8_2B). The allowance of scanning speed minus the reading speed was smallest for PD patients than in normal controls and SCD patients for all texts (Figures 7C_2C, 8C_2C). Fixation, saccade, and gaze durations varied slightly across text readability but were comparable among different subject groups for all texts (Figures 9C,D,E, 10C,D,E; restricting the analysis to MSA patients Figures 9_2C,D,E, 10_2C,D,E). The proportion of regressive saccades was slightly higher in SCD patients across all text readability than in normal subjects and PD patients, and decreased with decreasing readability of the text, with no significant difference between groups (Figures 9F, 10F; Tables 4, 5; restricting the SCD analysis to MSA patients Figures 9_2F, 10_2F; Tables 4_2, 5_2). The gaze would go well ahead of the uttered text for texts that were difficult to read, such that regression occurred only infrequently to compensate for this.

### EVL as an index of text readability and advance text processing

EVL became smaller as the text became more difficult to read (Tables 4, 5; restricting the SCD analysis to MSA patients Tables 4_2, 5_2). As the readability decreased and the reading speed was reduced, EVL also decreased. There was a significant interaction between groups and text for texts 20–22. This indicated that, although overall EVL was comparable among the three subject groups for most texts, with the exception of text 22 (Japanese syllabary), for which EVL was smaller in SCD patients in comparison to PD patients and normal subjects (Figures 7_2D, 8D; restricting the SCD analysis to MSA patients Figures 7, 8_2D).

**Figure 11:**
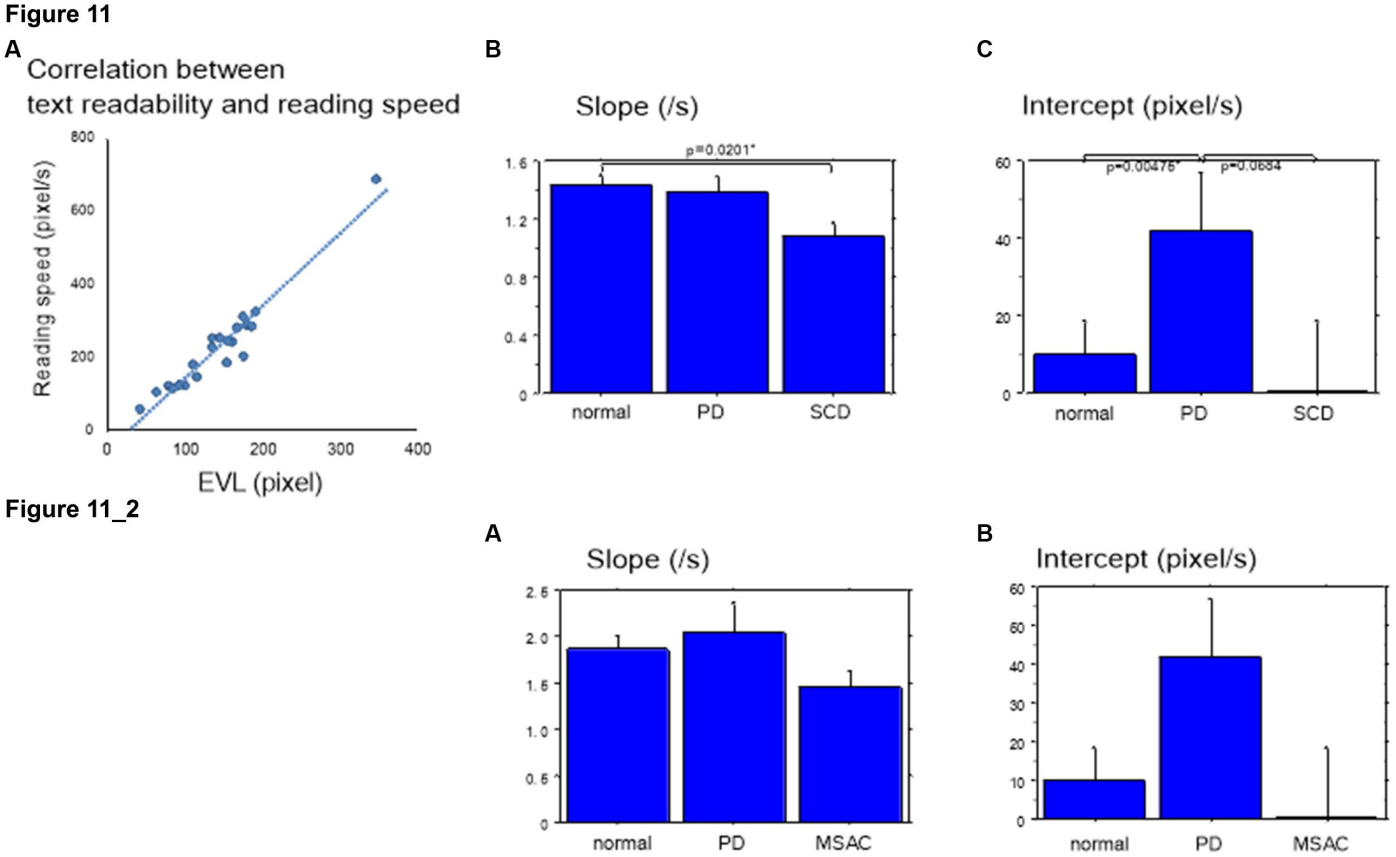
Slope and intercept of the linear correlation for reading speed as a function of EVL in the three groups of subjects. **(A)** Typical plot showing the correlation between spatial EVL (in pixels) and reading speed in a normal subject. **(B)** Slope, **(C)** Intercept of this correlation in different subject groups. Error bars show standard errors. Normal: normal subjects; PD: patients with Parkinson’s disease; SCD: patients with spinocerebellar degeneration. Asterisks indicate significant difference at *p < 0.05. **11_2** (lower two figures): Slope and intercept of the linear correlation for reading speed as a function of EVL in the three groups of subjects. Similar figures with conventions as in Figure 11 when the analysis of SCD patients were restricted to MSAC patients. **(A)** Slope, **(B)** Intercept of this correlation in different subject groups. MSAC, multiple system atrophy cerebellar-type.

EVL has been shown to correlate with reading speed, which is related to the automaticity or speed of reading (see Introduction). Conversely, EVL decreases with increasing text difficulty or decreasing text readability. Across different subject groups, EVL showed a moderate to strong correlation with reading velocity across text readability and across all texts (normal *r* = 0.79 ± 0.02, SCD *r* = 0.78 ± 0.04, PD *r* = 0.64 ± 0.08; Figure 11A). The slopes of this correlation (reading speed vs. spatial EVL) was small in SCD patients compared to normal subjects, but was comparable for PD patients and normal subjects (Figure 11B). This trend persisted when restricting the analysis of SCD patients to MSAC patients, although the difference between normal vs. SCD and PD vs. SCD failed to reach significance (Figure 11_2A; effect of group: *F*(2,124) = 4.261, *p* = 0.0189; *post hoc* analysis normal vs. SCD *p* = 0.0057, normal vs. PD *p* = 0.6256). Meanwhile, the intercept of the correlation was significantly higher for PD patients than for SCD patients and normal subjects across all texts (Figure 11C). The same was true when restricting the SCD patients to MSAC patients, although again, the difference between normal vs. SCD and PD vs. SCD failed to reach significance (Figure 11_2B; effect of group: *F*(2,124) = 4.887, *p* = 0.0109; *post hoc* analysis normal vs. PD *p* = 0.0033, PD vs. SCD *p* = 0.0055). This suggested that, in PD patients, reading speed was faster already at a small EVL, but they improved by a smaller amount with increasing EVL compared to normal subjects. In SCD patients, reading speed was comparable to normal subjects at smaller EVL levels, but again improved by a similar amount with increasing EVL than in normal subjects. As a result, the reading speed of SCD patients was smaller for all text readability than the other two groups.

**Figure 12:**
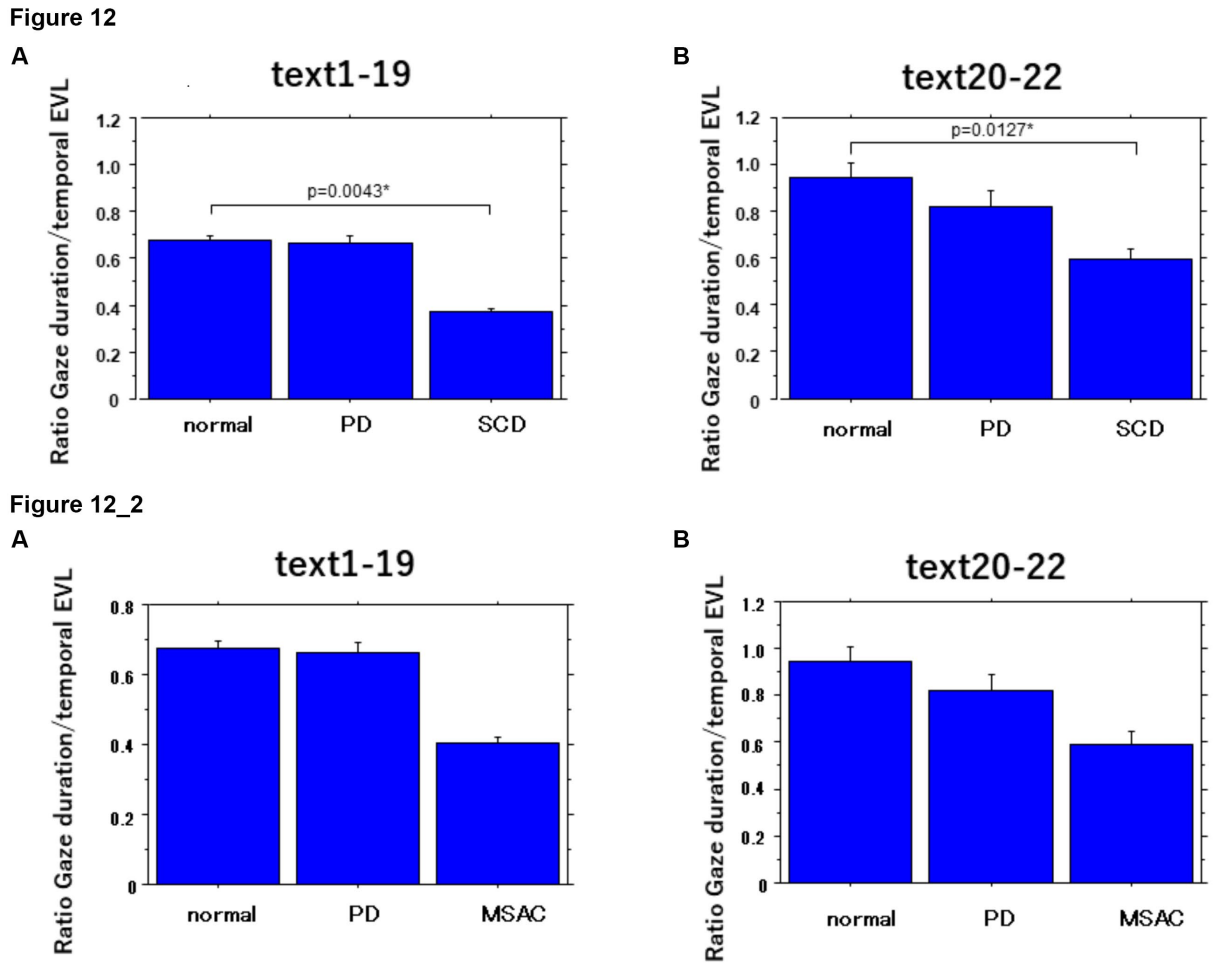
Ratio of gaze duration to temporal EVL in the three subject groups. Error bars show standard errors. Normal: normal subjects; PD: patients with Parkinson’s disease; SCD: patients with spinocerebellar degeneration. Asterisks indicate significant difference at *p < 0.05. **12_2** (lower two figures): Ratio of gaze duration to temporal EVL in the three subject groups. Similar figures with conventions as in Figure 12 when the analysis of SCD patients were restricted to MSAC patients. MSAC, patients with spinocerebellar degeneration.

The ratio of gaze duration/temporal EVL, a measure reflecting the weight of different processing stages (gaze-dependent vs. gaze-independent) within the onset EVS (Silva et al., 2016; see *Data Processing* section in the Methods), and thus the degree of parallel processing of the currently fixated word with the following word, was significantly smaller in SCD patients compared to other groups of subjects, while the difference between PD and SCD patients reached a trend but failed to reach significance for texts 1–19. This indicated lesser gaze-dependent factor in SCD patients (Figure 12; *post hoc* analysis: texts 1–19: normal vs. SCD *p* = 0.0043, SCD vs. PD *p* = 0.0293, texts 20–22: normal vs. SCD *p* = 0.0127). However, the difference between normal subjects and MSAC patients and between PD and MSAC patients did not reach significance when restricting the SCD patients to MSAC patients (Figure 12_2).

## Discussion

### Eye–voice coordination in reading aloud in normal subjects

When Japanese texts are read aloud by normal subjects, the gaze position in the text is consistently located spatially before the reading position by about 1 to 4 letters for all groups studied (spatial EVL). This is in contrast to the findings of Buswell (1921) and De Luca et al. (2013) in Western languages, where the EVL was reported to be between 12 and 18 letters. The discrepancy in EVL may be attributed to the difference between Western and Japanese writing systems. Japanese writing consists of a combination of hiragana (phonograms) and Chinese characters, with each phonogram generally corresponding to two or more letters in Western languages, and even more letters for Chinese characters. Additionally, Japanese texts typically do not have spaces between words. On the other hand, the temporal EVL, which refers to the time lag between gaze and reading position, is approximately 0.5–1.0 s ahead, consistent with previous findings in Western languages (as observed by Inhoff et al., 2011).

Even in the absence of the explicit segmentation of words and spaces in between in Japanese texts, stable eye-voice correlation with similar spatial and temporal EVL was observed for texts that consisted only or predominantly of hiragana, or texts consisting a mixture of hiragana and Chinese characters, with the latter possibly serving as a marker for the beginning of a word.

EVL was relatively stable throughout the texts, except where line changes took place, but varied depending on the overall readability of each text [text grade (Table 2)]. As the text became difficult to read (i.e., text grade increased), both the reading speed and the spatial EVL decreased and there was a significant correlation between them, consistent with previous reports that text reading automaticity correlates with text reading speed (Silva et al., 2016); enlarging EVL would serve to leverage the reading speed.

Laubrock and Kliegl (2015) suggested that the changes in text scanning speed with text difficulty are primarily modulated by saccade amplitude rather than saccade frequency, since saccade frequency remains relatively stable across different texts. In our study, when the reading speed is significantly slowed, individuals’ gaze almost remained fixated at a similar position or even make regressive eye movements toward the left. This occurred in texts in which graphemes were arranged in a randomized order, limiting the ability to process the text ahead of the gaze or chunk it into words or phrases. In such cases, the average reading speed dropped to 1.2–2.3 letters, whereas for other texts, it ranged from 2.3 to 3.7 letters. Even in these difficult texts, reading approached, if not reached, a “letter by letter” level, in which EVL is reduced down to a single letter level (Figure 8).

### Reading speed in PD and SCD patients

According to Figure 2, the reading position over time showed a flatter slope for SCD patients compared to the other two groups. This indicates that SCD patients exhibited a slower reading speed regardless of the readability of the text. The slower reading speed can be attributed to the impact on vocal output processing, affecting articulation in these patients. Even when presented with texts that had spaced intervals, such as text 22, SCD patients were unable to increase their reading speed to match those of normal subjects or PD patients. Additionally, the reading speed in SCD patients decreased as the severity of the disease progressed.

We expected the reading speed of PD patients to be slowed as well, considering the bradykinesia observed in these patients. However, reading speed was actually comparable or even be slightly more accelerated compared to normal subjects for texts with spaced intervals between words or phonograms (texts 20–22), nor did it decline with disease stage. Parkinsonian speech is characterized by hypophonia and dysprosody, sometimes with hastening, which worsens with disease progression, but not with slowness of vocal output (Skodda and Schlegel, 2008; Waldthaler et al., 2018; Stock et al., 2020).

### Interaction between text scanning speed and reading speed in PD and SCD patients

How does the altered gaze behavior in PD and SCD patients affect the eye–voice coordination during reading? In SCD patients, the speed of gaze scanning the text, excluding regressions, was only slightly slower than normal, contrasting with the reading speed that was much slower. As a result, the gaze could sometimes go well ahead of the voice, especially at the beginning of texts, such that regressions occurred frequently to keep pace with the slowly proceeding voice for most texts. Frequent occurrence of regressions may hamper advance text processing ahead of the gaze, degrading the visual input, and may lead to various reading impairments in SCD, such as letter reversals (adjacent, non-adjacent), letter insertion, word omission, addition, and verbal substitution (Moretti et al., 2002, 2003a,b). During visual search, patients with hereditary pure cerebellar ataxia exhibit a larger number of repeated fixations (re-fixations) of the target compared with normal subjects, because SCD patients may be unable to properly process and interpret what they are seeing on the first try (Matsuda et al., 2014).

Silva et al. (2016) reported a higher weight of different processing stages (gaze-dependent vs. gaze-independent) within the onset EVS, with a higher ratio associated with more weight in the gaze-dependent process. Namely, a higher ratio indicated more contribution of the parallel processing of words N (word fixated) and N + 1 (word to the right of the word fixated) to the complete processing (naming latency) of a given word N. In our study, the ratio of gaze duration to temporal EVL was lowest for SCD patients compared to the other two subject groups. This suggests that less parallel processing was taking place in SCD patients with less weight in the gaze-dependent process.

In contrast, PD patients exhibited almost normal reading speed. The frequency of saccades was significantly reduced in PD patients compared to normal subjects and SC patients, leading to significantly lower scanning speed. This may represent an oculomotor correlate of akinesia/bradykinesia. Consequently, the scanning speed of PD patients, on average, barely exceeded the reading speed in PD patients for all texts, and could not speed up further. The allowance for scanning gaze to precede the reading position was thus smaller for these patients, even for easy-to-read texts. Frequently, the gaze of PD patients scanning the text would even lag behind the read position of the text (Figure 1). The ratio of gaze duration to temporal EVL was comparable to normal subjects, indicating that parallell processing of words N and N + 1was was taking place as usual.

### Regulating reading speed by varying EVL for texts with various text readability

Similarly to normal subjects, the basic structure of eye–voice coordination was preserved in both SCD and PD patients, with the gaze preceding the voice by an approximately similar fixed amount of spatial separation (spatial EVL). Furthermore, PD and SCD patients as well as normal subjects exhibited a preserved ability to systematically modulate EVL according to text readability, and to keep this value relatively stable within each text reading.

EVL asymptoted to a certain level for easier texts, and became comparable among the three subject groups at around 2.5–4 letters (Figures 8, 9), where the maximal memory buffer size would have been reached. Even though the gaze theoretically could have gone farther ahead of the voice, subjects would have avoided overloading the memory load of the verbal sketchpad by restricting EVL within some range. At the other extreme end of text readability, in which hiragana was arranged in a randomized order, the letter to follow was unpredictable and reading was least “automatic.” For these texts, reading approximated “letter by letter” in all three subject groups and EVL became minimal at a single letter level, that is, the gaze would be at almost the same position as the uttered text.

In previous studies, EVL (eye voice lead) and EVS (eye voice span) have been found to be correlated with the automaticity of reading. Faster and more automatic reading is associated with a larger EVL size. The speed of naming during tasks like RAN is also influenced by EVS, but this correlation is observed mainly in highly automated processes such as digit naming, but not in less automated processes like dice naming (Pan et al., 2013; Silva et al., 2016).

In our current study, we investigated the relationship between reading speed and spatial EVL for different groups of participants. We found a significant correlation between reading speed and EVL in each subject group. However, the slope of this correlation, which represents the increase in reading speed with larger EVL, was lower in SCD patients compared to other groups. This suggests that SCD patients were unable to fully utilize a strategy of leveraging EVL to increase reading speed due to their inherent difficulty with fast articulation; despite their ability to move their gaze ahead of the reading position, they could not effectively employ this leverage strategy.

On the other hand, the intercept of the correlation (the starting point) was higher for PD patients with compared to both normal subjects and SCD patients. This indicates that even though PD patients had limited potential for increasing reading speed by “stretching” their EVL, they could still accommodate an EVL of 2.5–4 letters, as mentioned earlier.

Our research indicates that individuals with SCD encounter obstacles when it comes to effectively utilizing EVL for faster reading. This is primarily due to their difficulty in swift articulation. On the other hand, PD patients, despite their limited scanning speed, are able to operate within a defined range of EVL and sustain their reading speed. The difference in eye-voice coordination may also impact verbal processing. In SCD patients, the gaze sometimes moved well ahead of the voice, leading to more regressions and less parallel processing of words. This suggests that SCD patients had difficulties integrating word N + 1 while processing word N, with the gaze-dependent process playing a lesser role in their reading comprehension.

Paradoxically, when presented with a list of words arranged with spaces in between (Text 22, consisting of a Japanese syllabary with phonograms arranged in a predetermined order that the subjects had already memorized). PD patients actually showed a slightly faster reading speed compared to patients with SCD patients and normal subjects. This finding suggests that the reading speed itself was not inherently slowed in PD patients, but in some cases, it can even be potentially accelerated. PD patients may have utilized the spaced words or phonograms as external cues to enhance their reading speed, without the need for lexical processing of the phonograms. Interestingly, although the eye movements of PD patients were slightly delayed compared to their reading speed (as depicted in Figure 8C), this did not significantly hinder their overall reading performance.

### Limitations of the study

The study studied a limited number of subjects, particularly SCD patients, despite a recruitment period of over 5 years in a hospital that focuses on medical care for neurodegenerative disorders. The majority of SCD patients in the study presented with pure cerebellar manifestation or multiple system atrophy, which have distinct differences in their underlying causes. While both groups demonstrated a similar trend in eye-voice coordination, studying a larger number of subjects may reveal additional differences between these two types of disorders.

Another limitation would be the potential effect of medication, including levodopa and other dopaminergic drugs on the eye movement and vocal output, which cannot be completely discounted. We could not withdraw the medication completely due to ethical and clinical reasons. Although PD patients were examined approximately 3–4 h after drug intake which would have minimized the effects of l-dopa according to our previous studies, other dopaminergic drugs could not be completely washed out and we cannot exclude their persisting effects. In SCD patients, most of the drug taken was tartirelin hydrate, but there were some patients who were taking drugs that could have affected the central nervous system.

Both PD and SCD can affect eye movement and vocal output. Therefore, it is difficult to determine which of the two mainly affects eye-voice coordination. Our analysis showed that the relative contribution between these two were very different. The contrast between PD and SCD patients, with the scanning speed affected in the former and vocal output affected in the latter, allowed us to address the interaction of voice output and eye movements to scan the text.

Finally, the experimental design we used did not take into account certain intrinsic factors that could have influenced the correlation between eye movements and voice utterances. For instance, we did not compare reading aloud to silent reading, nor did we differentiate between reading meaningful and meaningless words, or between reading single words and reading complete sentences. Exploring these factors in future studies would provide valuable insights into the coordination between eye movements and voice outputs.

## Conclusion

EVL has been considered to reflect the size of the information included in the verbal sketch pad, a presumed biomarker for the amount of information that can be held in working memory while reading aloud. EVL was shown to be relatively intact in SCD and PD patients compared to normal subjects. Furthermore, these patients demonstrated the ability to adjust their reading speed by modulating EVL based on the demands of the text.

The process of reading aloud involves complex computations in different parts of the brain. It begins with converting a written letter string into a sequence of spoken sounds. Both familiar and unfamiliar words are processed using different pathways (Dehaene and Cohen, 2011). Processing of known words rely on the lexical route, which is influenced by word frequency. On the other hand, processing of novel words are constructed letter by letter through the sublexical route, which is sensitive to orthographic reading. Woolnough et al. (2022) conducted a study using fMRI to investigate the spatiotemporal map of reading aloud and identified a reading network involving several brain regions. This network includes the medial fusiform gyrus (mFus), inferior frontal gyrus (IFG), inferior parietal cortex (IPS), precentral sulcus, and the motor cortex. The findings suggest that reading involves simultaneous processing through the lexical route, from mFus (sensitive to word frequency) to IFG, and the sublexical route, from IPS and precentral sulcus to anterior IFG (sensitive to orthographic processing). The lexical route may support automatic and faster reading and enhance activity in the IFG and frontal eye fields, which are responsible for eye movement.

In this context, English is an alphabetic language, in which the sublexical pathway is primarily employed for reading regular words. However, learned irregular words may utilize the lexical pathway. On the other hand, reading Chinese characters heavily relies on the lexical pathway since the pronunciation of each character is learned through rote memorization; Chinese characters are logograms, which means they represent words or concepts directly, rather than individual sounds. Japanese text, however, represents a unique combination of phonograms (representing a phoneme) and morphemes (Chinese characters). This distinct composition makes Japanese a language that warrants further examination in future studies, allowing for a better understanding of the different use of lexical and sublexical pathways.

Instead of looking at the difference in character-based or phoneme/morpheme processing, here we looked at eye-voice coordination by changing the level of readability and looking at its impact on eye-voice coordination in PD and SCD patients. The interaction between eye movements and voice utterance differed in these two disorders, despite similar EVL comparable to normal subjects. While SCD patients experienced slowed eye movement scanning the text and vocal output, PD patients were able to maintain a relatively smooth reading process despite their inherent slowness in the text scanning speed. This finding holds potential significance as it highlights a distinct difference between PD patients and SCD patients in terms of eye-voice coordination during reading aloud. This discrepancy may serve as a potential disease biomarker for SCD and parkinsonism when reading aloud, although further research is needed to determine if it applies to all types of cerebellar ataxia and Parkinsonism disorders.

## Data availability statement

The original contributions presented in this study are included in the article/supplementary material. Further inquiries can be directed to the corresponding author.

## Ethics statement

The studies involving human participants were reviewed and approved by Ethics Committe of Graduate School of Medicine, University of Tokyo. The patients/participants provided their written informed consent to participate in this study.

## Author contributions

YT and S-iT performed the experiments, collected the data, and prepared the figures. YT wrote the main manuscript text and carried out patient recruitment. SI-T wrote the custom program for analyzing the data. All authors reviewed the manuscript and contributed to the discussion.

## Funding

YT was supported by a Research Project Grant in-aid for Scientific Research from the Ministry of Education, Culture, Sports, Science and Technology of Japan (18H05523) and Communications R&D Promotion Programme from the Ministry of Internal Affairs and Communications, Japan (B203060001). These funders were not involved in the study design, collection, analysis, interpretation of data, the writing of this article, or the decision to submit it for publication. YU received grants from the Ministry of Education, Culture, Sports, Science and Technology of Japan (Nos. 25293206, 15H05881, 16H05322, and 18K10821), Research Committee on the Medical Basis of Motor Ataxias, Health and Labour Sciences Research Grants, Ministry of Health, Labour and Welfare of Japan, Support Center for Advanced Telecommunications Technology Research, Association of Radio Industries Businesses and Novartis Foundation (Japan) for the Promotion of Science. S-iT was supported by a Research Project Grant-in-aid for Scientific Research from the Ministry of Education, Culture, Sports, Science and Technology of Japan (Nos. 19K17046, 21K15687).

## Conflict of interest

The authors declare that the research was conducted in the absence of any commercial or financial relationships that could be construed as a potential conflict of interest.

## Publisher’s note

All claims expressed in this article are solely those of the authors and do not necessarily represent those of their affiliated organizations, or those of the publisher, the editors and the reviewers. Any product that may be evaluated in this article, or claim that may be made by its manufacturer, is not guaranteed or endorsed by the publisher.

## Abbreviations

EVL: eye–voice lead
EVS: eye–voice span
RAN: rapid automatic naming
SCD: spinocerebellar degeneration
SCA: spinocerebellar ataxia
PD: Parkinson’s disease
ASD: autistic spectrum disorder
MSAC: multiple system atrophy cerebellar-type
ANOVA: analysis of variance
UPDRS: Unified Parkinson’s Disease Rating Scale
